# Ocean acidification boosts reproduction in fish via indirect effects

**DOI:** 10.1371/journal.pbio.3001033

**Published:** 2021-01-19

**Authors:** Ivan Nagelkerken, Tiphaine Alemany, Julie M. Anquetin, Camilo M. Ferreira, Kim E. Ludwig, Minami Sasaki, Sean D. Connell

**Affiliations:** Southern Seas Ecology Laboratories, School of Biological Sciences and The Environment Institute, The University of Adelaide, Adelaide, South Australia, Australia; Trinity CollegeDublin, University of Dublin, IRELAND

## Abstract

Ocean acidification affects species populations and biodiversity through direct negative effects on physiology and behaviour. The indirect effects of elevated CO_2_ are less well known and can sometimes be counterintuitive. Reproduction lies at the crux of species population replenishment, but we do not know how ocean acidification affects reproduction in the wild. Here, we use natural CO_2_ vents at a temperate rocky reef and show that even though ocean acidification acts as a direct stressor, it can indirectly increase energy budgets of fish to stimulate reproduction at no cost to physiological homeostasis. Female fish maintained energy levels by compensation: They reduced activity (foraging and aggression) to increase reproduction. In male fish, increased reproductive investment was linked to increased energy intake as mediated by intensified foraging on more abundant prey. Greater biomass of prey at the vents was linked to greater biomass of algae, as mediated by a fertilisation effect of elevated CO_2_ on primary production. Additionally, the abundance and aggression of paternal carers were elevated at the CO_2_ vents, which may further boost reproductive success. These positive indirect effects of elevated CO_2_ were only observed for the species of fish that was generalistic and competitively dominant, but not for 3 species of subordinate and more specialised fishes. Hence, species that capitalise on future resource enrichment can accelerate their reproduction and increase their populations, thereby altering species communities in a future ocean.

## Abstract 2

Ocean acidification affects species populations and diversity through direct negative effects on physiology and behavior, but the indirect effects are less clear. Using volcanic carbon dioxide vents as natural analogues of future ocean acidification, this study shows that elevated CO2 can stimulate fish reproduction in the wild through increased food abundance, leading to increased energy budgets at no cost to physiological homeostasis.

## Introduction

Resources are often limited in nature. They constrain the capacity of an environment to meet the energetic demands of organisms, thereby shaping the evolution of life history strategies that underpin their fitness and their population persistence [1,2]. Organisms have many competing demands of high-energy-consuming processes (e.g., maintenance, growth, and reproduction) and need to continually trade off their allocation of energy between them [3,4]. Future climate represents a major change to organisms as they adjust their behaviour [5] and physiology [6] to accommodate changes in resource availability [7]. Yet, we have little understanding of how these adjustments combine so that life history trade-offs alter an individual’s fitness and sustainability of their populations in a future world.

Although physiological and behavioural trade-offs in energy allocation and expenditure are exacerbated under chronic (e.g., climate) stress, these can be mitigated when animals have access to abundant food resources and do not need to rely on endogenous energy reserves [8]. Results from laboratory environments are therefore difficult to transfer to nature, because resource availability and species behaviours (e.g., energy-consuming activities like predator avoidance, mating, feeding, competition, and parental care) are key drivers of energy intake and expenditure in the wild, and such ecological complexity can buffer or exacerbate climate change impacts [9]. Furthermore, sex-based differences in response to ocean acidification have largely been ignored, even though genders have different involvement in and energy allocation strategies and costs towards reproduction [10]. As such, pooling gender-specific responses has created an average phenotype that does not exists in nature and leads to large inaccuracies in predicting how species populations as a whole will respond to ocean acidification [10].

Ocean warming increases metabolic rates in most ectotherms [6], while ocean acidification has negative effects on invertebrate prey abundances [7]. As a result, a mismatch between energy demands and energy availability in marine ectotherms is anticipated in a future warm and acidified ocean [7], with reduced energy flow propagating up higher trophic levels of marine food webs [11,12]. Ocean acidification further increases energy expenditure on very costly acid–base regulation in vertebrates [13]. With a concurrent reduction in resource availability and an increased energy allocation towards homeostasis under elevated CO_2_, reduced energy is predicted to be available for reproduction [4], ultimately affecting species population replenishment and thus biodiversity.

**Fig 1:**
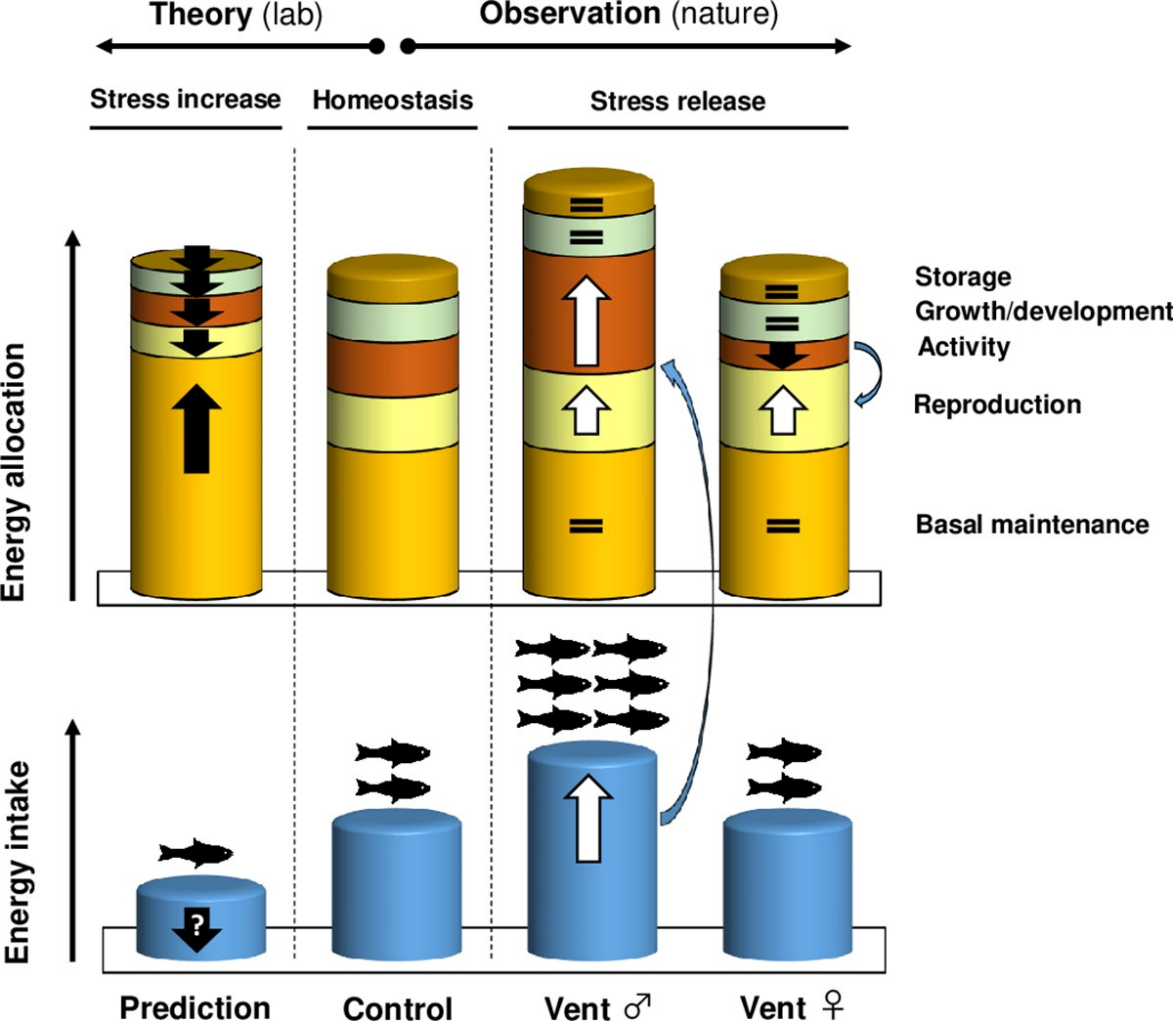
Relative energy allocation towards key physiological processes. The 2 left-hand columns represent our predictive stress model to ocean acidification (compare “homeostasis” vs “stress increase”) and have been adapted from [4]. The 3 right-hand columns represent the empirical findings of our study (compare “homeostasis” vs “stress release”). Sizes of the discs indicate the relative importance of various processes. Arrows indicate increases (upwards) or decreases (downwards) with white reflective of positive changes and black reflective of negative changes compared to homeostasis; ? indicates unknown. Under increased stress, energy allocation towards basal maintenance increases significantly due to the higher cost of protection against and damage repair from stress, with energy being reallocated from other processes; at the same time, reduced energy intake might occur due to stress conditions. The comparison between “homeostasis’” and “stress release” in female and male fishes at CO_2_ vents summarises our empirical observations. Females reallocate energy from activity to reproduction, while males use increased energy intake for increased reproduction and reproduction-related activity (inter-mature male aggression). The number of fish reflects changes in fish population sizes. Fish symbol from https://openclipart.org/.

We used 2 natural CO_2_ vents at White Island, New Zealand, to test the model that ocean acidification reduces fitness (i.e., via reproduction) by simultaneously reducing energy availability (i.e., reduced resource abundance) and increasing energy expenditure (i.e., costly acid–base regulation to maintain homeostasis) (Fig 1). We study 4 benthic, site-attached fish species in situ across 3 years in a temperate kelp–rocky reef system and evaluate differences among sexes. By using observations from the wild, we incorporate natural demographic processes, species interactions, animal behaviour, and ecosystem responses that span different levels of biological organisation—from cells, to organs, to organisms, to populations, to ecosystems. We reveal a novel model where increased prey resources under elevated CO_2_ boost energy budgets and can enhance individual fitness through increased reproductive investment and altered demography in fish.

## Results

**Fig 2:**
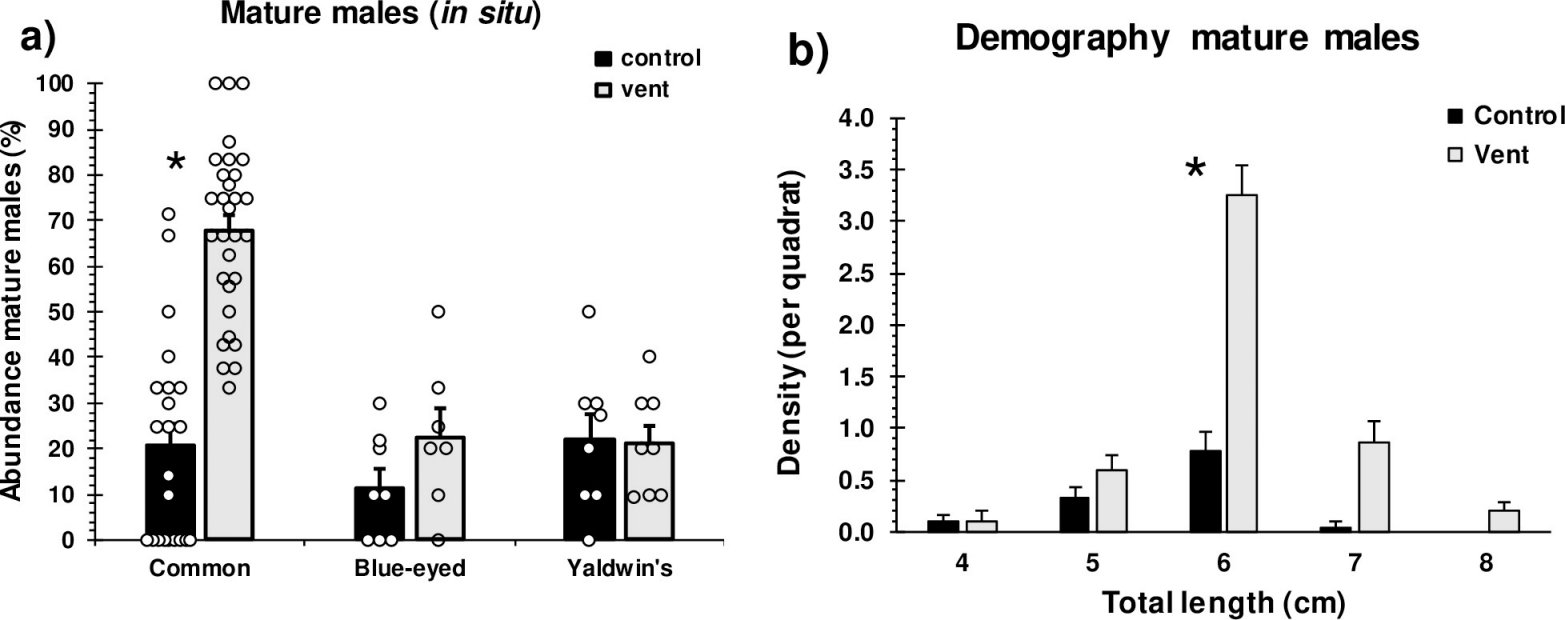
**Mean (+ SE) relative abundance of mature males of the common, blue-eyed, and Yaldwin’s triplefins, respectively (a), and size–abundance distribution of mature males of the common triplefin (b), at control and CO**_**2**_ **vent sites.** The abundance of mature males was quantified in situ based on gender-specific (nuptial) body colouration and calculated as the number of mature males relative to total number of males, females, and juveniles per transect; this was not possible for the crested blenny which lacks secondary sex characteristics. Data are from years 2017 to 2019 (S4 Table), with circles representing replicate transects (jittered on *x*-axis where values overlap; * significant difference (*p* < 0.05) between treatments (S1 Table). See S1 Data for the underlying data.

The most common fish species showed a >3-fold higher relative abundance of mature males at vents than controls across years (Fig 2A; 3-way analysis of variance (ANOVA), *p* = 0.001, S1 Table). These differences are explained by denser populations of large males at the vents (Fig 2B; 4-way ANOVA − treatment × size interaction, *p* < 0.001, S1 Table) and are indicative of higher survival rates of large males, as densities of smaller males did not differ with partial pressure of carbon dioxide (*p*CO_2_) levels. Faster growth rates did not explain the higher densities of large males, as there was no concurrent density decrease in smaller male size classes, total population sizes of males (i.e., all sizes pooled) were higher at the vents, and male growth rates were similar at vents and controls (S1A Fig). Juveniles and females showed no difference in size–frequency distribution between vents and controls (S1B Fig; *p* = 0.565). The other fish species did not show a higher relative abundance of mature males at vents than controls (Fig 2A, S1 Table).

**Fig 3:**
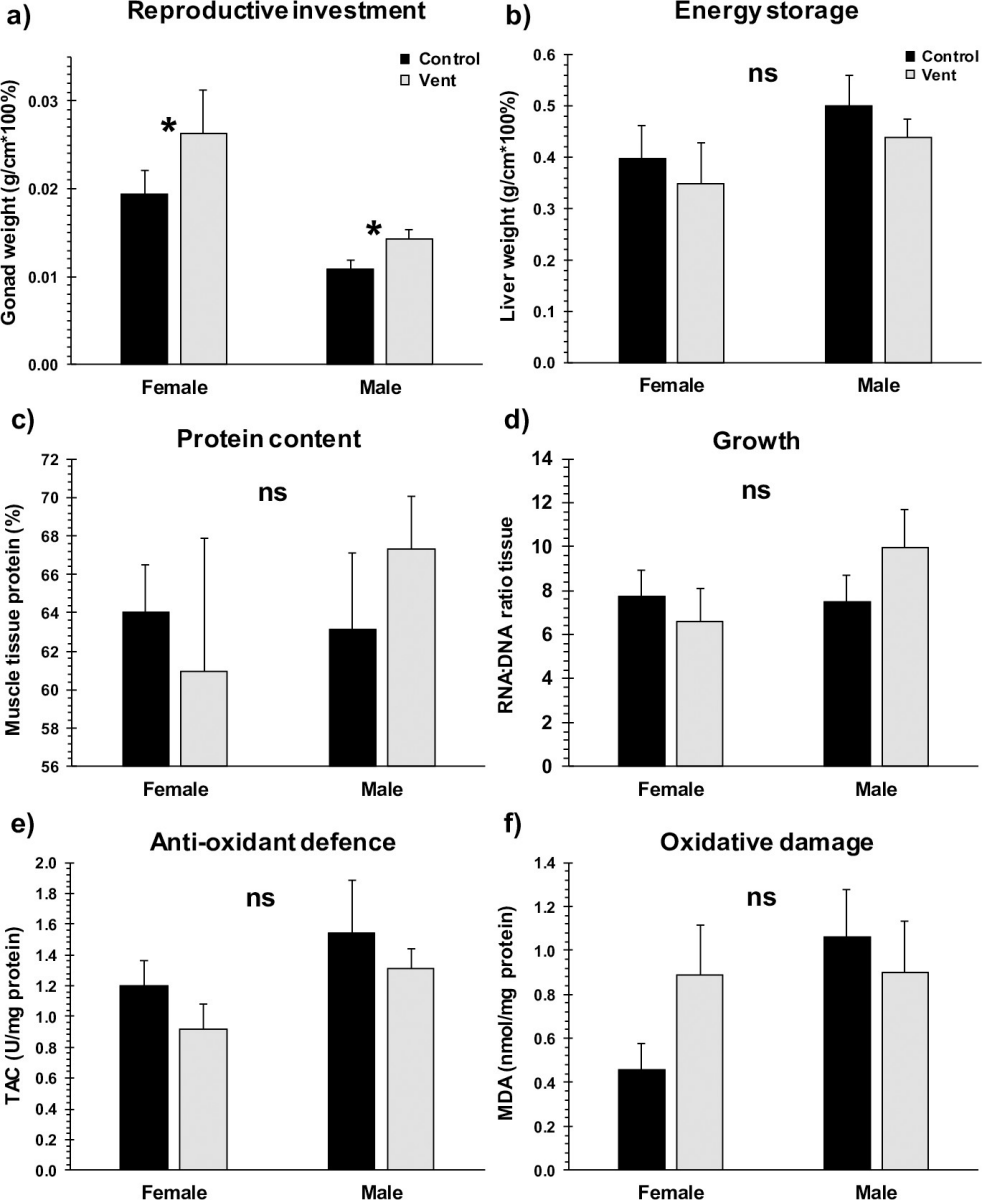
Physiological proxies for the common triplefins from control and CO_2_ vents. Mean (+ SE) relative gonad weight (**a**), relative liver weight (**b**), crude tissue protein content (**c**), tissue RNA:DNA ratio (**d**), tissue total antioxidant capacity (**e**), and tissue malondialdehyde levels (**f**), for female and male common triplefins collected from control and CO_2_ vent sites during 2017–2019 (S4 Table). Gonad and liver weights were both standardised as a function of fish total lengths. Data for the other fish species are presented in S2 Fig. * significant difference (*p* < 0.05) between treatments (S1 Table). See S1 Data for the underlying data. ns, not significant.

Although densities were skewed towards (mature) males at vents, both females and males of the most common species showed a higher investment in reproduction at vents (Fig 3A; 3-way ANOVA, *p* = 0.010, S1 Table), irrespective of significant gender effects (*p* = 0.006). The higher investment in gonads at vents did not occur at the detriment of other physiological traits (3-way multivariate analysis of variance (MANOVA), *p* = 0.814; Fig 3, S1 Table), in either females or males. The other 3 fish species did not show differences in their reproductive investment or any of the physiological traits at vents compared with controls (S2 Fig, S1 Table). Neither of the 4 species showed differences in sex ratio at vents vs controls (S1C Fig, S1 Table), and these species do not undergo sex change with age.

**Fig 4:**
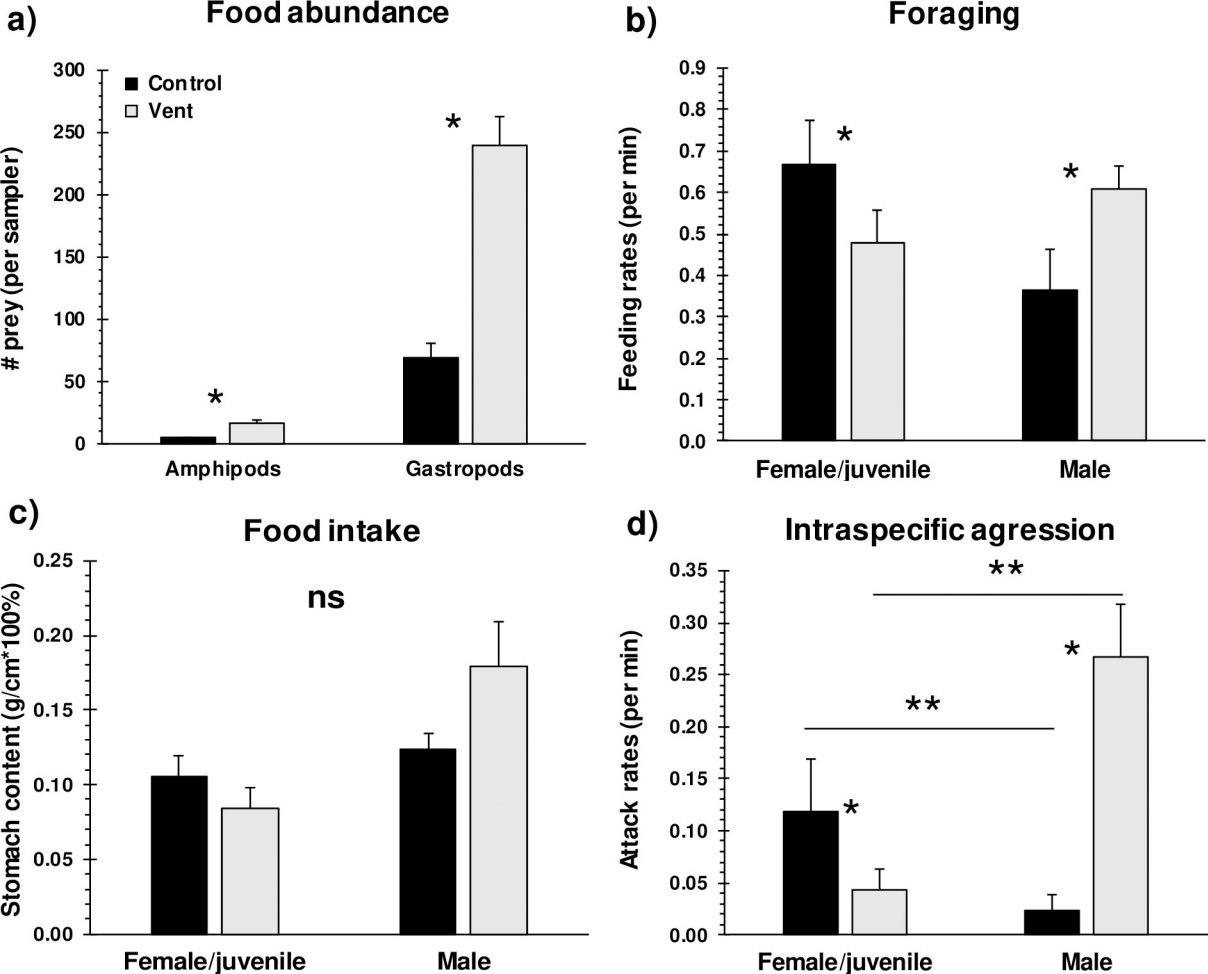
**Mean (+ SE) benthic food abundance (a), in situ feeding rates (b), total stomach content weights (c), and in situ intraspecific aggression rates (d) of mature male and female/juvenile common triplefin at controls and CO**_**2**_ **vents in 2017.** Intraspecific aggression refers to aggressive behaviours of females/juveniles and males towards their same gender, respectively. The weight of total stomach content was standardised as a function of fish total lengths. Data for the other fish species are presented in S5 Fig. * significant differences (*p* < 0.05) between control and vents (S2 Table); ** significant differences between males and females/juveniles within controls or vents, respectively. See S1 Data for the underlying data. ns, not significant.

Benthic prey abundances were 3.5 to 4 times higher at vents than controls (Fig 4A; 2-way MANOVA, *p* = 0.018, S2 Table). The biomass of benthic prey was positively correlated to primary production (S3 Fig; *R*^2^ = 0.64, *p* < 0.0001), with primary production being higher at vents than controls (2-way ANOVA, *p* = 0.029, S2 Table). Correspondingly, feeding rates (Fig 4B; 3-way ANOVA − treatment × gender interaction, *p* = 0.028, S2 Table) of mature males of the most common fish species were approximately 1.5 times higher at vents, although stomach fullness remained the same as at the controls (Fig 4C). Territorial aggression rates among mature males were >10-fold higher at vents than controls (Fig 4D; 3-way ANOVA − treatment × gender interaction, *p* = 0.027). Male aggression rates were positively correlated to mature male densities at controls (S4 Fig; *R*^2^ = 0.37, *p* = 0.038) but not at vents (*R*^2^ = 0.02, *p* = 0.550), even though mature male densities at vents reached up to double that of controls (S4B Fig). In contrast to males, females and juveniles showed lower rates of foraging (*p* = 0.002) and aggression (*p* = 0.031) at vents (Fig 4, S2 Table). Feeding rates, intraspecific attack rates, and stomach fullness of the other fish species did not differ between controls and vents, except for lower stomach fullness at vents for the blue-eyed triplefin (S5 Fig, S2 Table).

A piecewise structural equation model (SEM) did not identify any missing pathways or the probability that the pathways of the effect of CO_2_ enrichment on primary production, food abundance, and food intake occurred by chance, as supported by an acceptable goodness of fit (S5 Table). CO_2_ enrichment had a positive direct effect on primary producer biomass (*R*^2^ = 0.26, *p* = 0.027) and food abundance (*R*^2^ = 0.75, *p* < 0.0001) and either a direct (via feeding behaviour) or indirect (via increased food abundance) positive effect on food intake in male common and blue-eyed triplefins, in male and female Yaldwyn’s triplefins, and in female crested blennies (S6 Fig). Reproductive investment increased in male common triplefins under CO_2_ enrichment (*R*^2^ = 0.27, *p* < 0.006) and possibly also due to increased food intake (near significant *p* = 0.058). Increased food intake had positive effects on energy storage and antioxidant defence in several species, while several species appeared to trade-off energy allocation among reproduction, growth, energy storage, body condition, and maintenance. It should be noted, however, that many of the pathways were based on relatively low numbers of fishes and may not all be causal.

Heavy metal, trace element, and sulphur concentrations in seawater sampled from the fish-collection and video-observation quadrats did not differ between controls and vents across years (S3 Table) and were unrelated to the altered demography, reproduction, and behaviours at vents. Only pH and *p*CO_2_ differed between vents and controls. There was no direct effect of pH or temperature levels on the sex ratio or gonad weight (S7 Fig), except for a significant positive relationship between male gonad weight and decreasing pH (linear regression, *p* = 0.003) but with negligible explanatory power (*R*^2^ = 0.05).

## Discussion

Using natural CO_2_ vents as analogues of ocean acidification, we show that elevated CO_2_ can boost fish reproduction in the wild through complex pathways that operate at multiple levels of biological organisation. Our study suggests that boosted reproduction is mediated by indirect effects rather than direct effects of elevated CO_2_ on fish reproductive tissues. Ocean acidification altered no less than 3 reproductive traits at CO_2_ vents: investment into gonads by both genders, abundance of large mature males (parental caregivers), and territory/nest defence by mature males. These traits mediate reproductive output through different pathways, as discussed below.

Females and males of the most common species had greater gonad weight at the vents. Such investment into gonads is energetically costly and is often a lower priority relative to other physiological processes such as basic maintenance and growth in longer-lived animals [4]. However, our species have an opportunistic life history strategy, which is characterised by small body size, low trophic level, and short life span, but with relatively high fecundity and parental care [14]. Such opportunistic species typically prevail in unstable and unpredictable environments [15]. Males capitalised on the increased food abundance at vents by increasing their feeding rates (Fig 1). While stomach fullness of males did not differ between controls and vents, their foraging rates were higher at vents. Moreover, structural equation modelling revealed a significant positive relationship between elevated CO_2_ and food intake and reproductive investment, respectively. The surplus energy from increased feeding supported heavier gonads and increased territory defence at the vents, with the latter not related to the greater densities of mature males at vents. Mature males of the fish species studied here provide parental care up to 3 weeks by aggressively defending their small territories in which eggs are deposited by the females [16]. Nest defence and parental care are very costly processes, but the benefits can increase fitness through enhanced hatching, development, and survival rates [17]. While animals with parental care often face a trade-off between time spent defending their brood and feeding [18], mature males were able to increase both behaviours at the CO_2_ vents, likely because feeding is less time-consuming at elevated prey densities [19]. We conclude that increased parental aggression—fuelled by enhanced energy budgets, combined with a higher abundance of parental caregivers (i.e., mature males) at vents—could stimulate reproductive output through increased brood success under elevated CO_2_.

Females did not increase their energy budgets through increased feeding at the vents and therefore appear to trade off activity levels (foraging and aggression) for increased reproduction (Fig 1). However, reduced foraging under higher prey abundances allowed similar levels of food intake and therefore maintenance of homeostasis under elevated CO_2_. Increased investment into egg production provides a greater boost to fitness than increased sperm production as sperm is seldom limiting [20]. Hence, for species that provide parental care, increased egg production by females combined with increased nest defence by males creates a combined gender-specific mechanism that allows for boosted reproductive output under elevated CO_2_.

In contrast to predictions, all 4 species maintained homeostasis under elevated CO_2_. Laboratory studies have shown variable results (negative, positive, or no effects) of ocean acidification on oxidative status, condition, energy storage, or growth in a range of fishes (e.g., [13,21–23]), although longer-term studies indicate that species might be able to minimise effects at cellular levels through acclimation [24]. Fishes collected from the vents, having been exposed to elevated CO_2_ for the majority of their life cycle, did not show significant changes in bioenergetic markers of stress at cellular or organ levels (i.e., RNA:DNA ratio; total antioxidants; oxidative damage, and protein levels in muscle tissue, respectively; and energy storage in livers). Instead, the single common species that increased its energy budget (males) or reallocated its energy (females) at the vents showed increased reproduction. Hence, increased energy budgets and altered energy allocation strategies provide opportunities for some species to increase their fitness under ocean acidification.

The alterations in fish physiology and behaviour at the vents are most likely caused by elevated CO_2_ rather than other drivers (Table 1). Seawater temperature which is known to regulate reproduction [25] was not altered at the vents. Differences between vents and controls were not confounded by sulphur or heavy metals. Ocean acidification has been found to directly stimulate reproduction (number of egg clutches and number of eggs per clutch) under laboratory conditions in tropical and temperate fish species [22,26,27], possibly due to alterations to the endocrine system [13,26]. However, we observed only a very weak correlation between pH levels and female or male gonad weights or between pH and sex ratio. We therefore conclude that in the wild, ocean acidification has stronger positive effects on fish reproduction via indirect pathways (i.e., increased energy budgets, altered energy allocation strategies, altered demography, and enhanced reproductive-related behaviour) rather than direct effects on gonad development (Table 1).

**Table 1:** Hypotheses that could explain the observed increase in reproductive investment (gonads) for the most common benthic fish (common triplefin) at CO_2_ vents.

Hypothesis	Supported	Supporting observation
Direct effect of pH on gonads (e.g., via endocrine system)	No (?)	Only a very weak correlation between fish gonad weight and pH levels
Energy reallocation	No (♂)	Mature males showed similar energy storage, protein content, energy allocation towards growth, growth rate, cellular defence, and oxidative damage at controls vs vents. SEM did not identify negative correlations among physiological traits
Energy reallocation	Yes (♀)	Females showed lower activity levels (foraging/aggression) but larger gonads at vents vs controls. SEM identified a weak negative correlation between reproduction and body condition
Indirect ecosystem effects	Yes (♂)	Elevated CO_2_ increased primary production, which increased prey abundances and biomass, increasing foraging rates in mature males
Indirect ecosystem effects	No (♀)	Elevated CO_2_ increased prey abundances, but reduced foraging rates, and therefore, food intake remained unaltered in females
Vent contamination	No	Heavy metal and trace element concentrations did not differ between control and vent fish collection sites
Elevated seawater temperature	No	No correlation between fish gonad weight and seawater temperature

SEM, structural equation modelling.

In contrast to ocean warming, elevated CO_2_ often has positive effects on marine food webs through bottom-up CO_2_ enrichment [12,28]. Increased primary productivity and primary producer biomass under ocean acidification enhances food availability to both vertebrate and invertebrate herbivores [29,30], whose increased abundances and productivity fuel abundances at higher trophic levels [31]. While increased productivity is frequently observed at multiple food web levels, studies suggest that generalists, or “weedy” species, benefit most from this resource increase due to their life history characteristics, including wide and flexible trophic and habitat niches [32,33]. This allows generalists to quickly adapt to a changing environment to the detriment of more specialist species. Our most common fish species is not only a habitat generalist [34] but also bolder, more aggressive, and more competitive in feeding behaviour than the other 3 species at the vents [35], which may explain why greater reproductive investment was only observed in this behavioural dominant. Hence, increased resource availability provided greater benefits to a competitive and generalist species that can capitalise on the increased energy provision by fuelling this energy towards increased reproductive output.

It is unclear if increased reproductive investment alone will ultimately lead to higher species population replenishment in a future ocean, because young life stages are particularly vulnerable to environmental change [36]. The greater vulnerabilities of earlier life stages of larvae to CO_2_ enrichment have been shown in the laboratory and natural upwelling areas, particularly for fitness-related traits of growth and condition [37], survival [38], hiding behaviour [39], and the antioxidant defence system [21]. Indeed, an integrative model (incorporating laboratory results on egg fertilisation, egg and larval survival, and development time) forecasts recruitment failure for Atlantic cod under ocean acidification and warming, but also suggests that future reductions in recruitment success might be mitigated via increased food availability, adaptation, and increased egg production (through fisheries stock management), with egg production having the strongest buffering effect [40]. Food availability increases for some species under elevated CO_2_ (this study), while adaptation/acclimation on the short term might be accomplished through parental effects [41] or acclimated populations that are already experiencing naturally high fluctuations in *p*CO_2_ [42]. Moreover, the survival of early life stages might increase due to lower abundances of some predators under elevated CO_2_ [43,44]. Therefore, any increased fitness-related sensitivity to ocean acidification during the early larval stage might be offset by increased food abundance, lower predation, and adaptation, combined with increased reproductive output and parental care as revealed in our study.

Early life stages are considered most sensitive to ocean acidification, yet we reveal 2 mechanisms that can buffer its negative effect on populations: increased reproductive investment by parents and increased abundance and aggressiveness of caregiving parents at CO_2_ vents. We conclude that energetic trade-offs by egg-producing females and increased energy budgets and abundances of male caregivers can together stimulate reproductive success of some species in a high-CO_2_ world.

## Methods

### Ethics statement

All experiments were performed under animal ethics approval numbers S-2015-222 and S-2015-019 by the University of Adelaide Animal Ethics Committee, in accordance with the South Australian Animal Welfare Act 1985 and in compliance with the Australian Code for the care and use of animals for scientific purposes (8th Edition 2013).

### Study site

White Island is a volcanic island located in the Bay of Plenty of the North Island of New Zealand. Two independent vent and 2 control sites were identified along the northeastern coast of the Island. The CO_2_ plumes at vent sites were approximately 24 × 20 m in dimension and located at 6 to 8 m depth. The control sites were located adjacent to the vents (approximately 25 m away), and pH levels represented ambient oceanic conditions (S3 Table, mean range across years: 8.03 to 8.08). The vent sites had reduced seawater pH (mean range across years for vents combined: pH = 7.83 to 7.88, ΔpH control − vent = 0.17 to 0.25). The southern vent showed a mean pH reduction of 0.17 pH units compared with the control site, reflecting an approximate representative concentration pathway (RCP) 4.5 scenario for year 2100 with a forecast ΔpH = −0.15 [45], while the northern vent showed a mean pH reduction of 0.24 pH units which is close to an RCP 6.0 scenario with a forecast ΔpH = −0.22 [45]. The pH levels at the 2 vent sites were not confounded by higher temperatures (S3 Table). The study area represents a hard substratum rocky reef ecosystem, and the substratum at control sites was characterised by a mosaic of kelp (*Ecklonia radiata*), turf-forming macroalgae (<10 cm in height), and hard substratum sea urchin barrens devoid of vegetation [44].

### Seawater carbonate chemistry

During February 2017, water temperature and pH levels were measured inside each individual fish quadrat at the same time as the respective fish surveys (approximately 15 cm above substratum), while water samples for total alkalinity and salinity were randomly collected in 2017 from control and vent sites (S3 Table). During 2018 and 2019, all water samples (for pH and salinity) and all water temperature measurements were taken randomly across the control and vent sites at approximately 50 cm above the substratum. For all 3 years, the water temperature was measured in situ for approximately 30 min using a Hobo Pendant Temperature Data Logger (8K-UA-001-08; Onset, Massachusetts). For all years, pH of the water samples was measured within 30 min after collection using a SevenGo SG2 pH metre (Mettler Toledo, Victoria). Salinity was measured with a Vital Sine SR6 refractometer (Pentair, Florida) in 2017 and with an Ohaus ST20S starterpen salinity metre in 2018 and 2019. Alkalinity was measured only in 2017 for water samples taken from the water column (approximately 1.5 m above substratum). Alkalinity water samples were fixed with mercuric chloride and preserved in Duran glass bottles (DWK Life Sciences, Mainz) until analysis, according to standard operating procedures [46]. Total alkalinity was measured by dynamic endpoint titration using a Titrando (Metrohm, Herisau) titrator. Values for standards were successfully maintained within 1% accuracy from certified reference materials from Dr A. Dickson (Scripps Institution of Oceanography). The *p*CO_2_ concentration of each water sample was calculated using the respective values of temperature, salinity, pH_NBS_, and total alkalinity. The software CO2SYS was used to estimate seawater *p*CO_2_ with constants K1 and K2 from [47] and refit by [48].

### Seawater trace elements

During February 2018 and January 2019, between 7 and 10 water samples were collected approximately 0.5 m above the substratum at control and vent sites for in-depth water chemistry analysis (S3 Table). Samples were immediately preserved after collection with hydrochloric acid (HCl) to a final concentration of 3% HCl. In the laboratory, the seawater samples were diluted with deionised water (50 times or 2,000 times) to ensure that the concentrations of elements were within the detection range of the inductively coupled plasma mass spectrometer (ICP-MS) (Agilent 7500cs, Agilent Technologies, USA). Concentrated HCl was added during the dilution process so that the diluted samples remained at a concentration of 3% HCl. Using an ICP-MS, the concentrations of barium (Ba), cadmium (Cd), calcium (Ca), copper (Cu), iron (Fe), magnesium (Mg), manganese (Mn), potassium (K), rubidium (Rb), strontium (Sr), sulphur (S), and zinc (Zn) were determined. Procedural blanks (i.e., deionised water in 3% HCl) were also included in the analysis (5 samples per year). The concentrations of all above elements in the blanks were below detection limit, indicating the lack of any contamination of the deionised water used for dilution.

### Fish surveys

A limitation of using natural CO_2_ vents to represent future acidified ecosystems is that many animals move in and out of the vent areas and are therefore not continuously exposed to elevated CO_2_. To work within this limitation, we focussed on highly site-attached species (triplefins—Tripterygiidae—and blennies—Blenniidae) that occupy territories directly after settlement, show little movement, and have a small home range of just 1–2 m^2^ [16,49]. Triplefins reach their highest global diversity in New Zealand. They live for approximately 2 to 2.5 years of age and die after having participated in 2 breeding seasons [50]. Mature males provide parental care within their territories for 12 to 20 days, through nest defence against predators, oxygenating of eggs through fanning, and keeping eggs clear of siltation [16]. When triplefin males are actively caring for a nest they show a distinct nuptial colouration [50]. During the first study year (2017), we focussed on the most abundant benthic fish species at the study site, the common triplefin *Forsterygion lapillum*. This species accounted for 67% and 90% of the total benthic fish density at control and vent sites, respectively [35]. Males turn from yellow to black in colouration during their spawning and breeding activity, while immature males and all females remain yellow in colouration [50]. We also included the next 2 most common triplefin species: blue-eyed triplefin (*Notoclinops segmentatus*) and Yaldwin’s triplefin (*Notoclinops yaldwyni*). Breeding males of these 2 species show a change in colouration of their 9 vertical body bands from red to black (blue-eyed triplefin) and a bright orange body colouration (Yaldwin’s triplefin), respectively [50].

To determine the abundance of breeding adults, the total number of individuals (S4 Table) and number of mature males (common, Yaldwin’s, and blue-eyed triplefins, respectively) were visually quantified using SCUBA during February 2017 in replicate of 0.5 × 0.5 m polyvinyl chloride (PVC) quadrats (32 in total) at the 2 vent sites (10 and 10 replicate quadrats, respectively) and 2 control sites (6 and 6 replicate quadrats, respectively). The quadrat was randomly placed on the substratum from which the fishes did not swim away during its deployment. For the common triplefin also, the total length of each yellow and black individual was visually estimated. Directly upon completion of each visual count, a water sample for pH measurement (see Seawater carbonate chemistry) was collected and a temperature logger (see Seawater carbonate chemistry) and GoPro camera (see Fish behaviours in situ) were deployed inside each quadrat.

During January 2019, surveys to quantify the abundance of breeding males were repeated. Total length, colouration (black breeding males versus yellow females/juveniles), and number of individuals (S4 Table) of the common triplefin were quantified in 5 replicate 0.5 × 0.5 m PVC quadrats at each of the 2 vent sites (20 quadrats in total). The Yaldwin’s and blue-eyed triplefins were not common enough to be sufficiently sampled in these quadrats, and hence, roving transects were employed. The benthic substratum was intensively searched for these 2 species, and each set of 10 consecutive individuals encountered (per species, S4 Table) was allocated to a replicate roving quadrat. In total, 3 replicate roving quadrats were performed at each of the 2 control and vents sites (12 quadrats in total), for each species. For each individual of each of these 2 species, total length, and colouration (breeding males versus females/juveniles) were quantified.

### Fish behaviours in situ

During 2017, fish behaviours were quantified from video recordings taken inside the same replicate 0.5 × 0.5 m PVC quadrats that had been used to quantify the abundance of mature males and females/juveniles in situ. Directly after completing the visual fish counts, a GoPro camera was placed at the border of the quadrat and left to record the entire quadrat for 10 min without the presence of a diver. A total of 35 recordings were taken (S4 Table), which included the same 32 quadrats used for visual census (see Fish surveys) plus 3 additional quadrats in which no fish census had been performed. The Yaldwin’s and blue-eyed triplefins did not occur in every video recording, and hence, data from 19 and 12 recordings, respectively, were available for these 2 species (S4 Table). The crested blenny was not observed in any of the 38 video recordings.

Subsequently, for each video recording, the number of foraging bouts and the number of attacks on conspecifics was quantified for each individual fish inside the quadrat and expressed as a rate per minute. Attacks represented aggressive chasing of another fish. For the common triplefin, we quantified intra-gender attacks (male versus male and female/juvenile versus female/juvenile). Because it was difficult from the recordings to distinguish mature males from females/juveniles in the blue-eyed and Yaldwin’s triplefins, we only counted intraspecific attacks for these 2 species. Other fish species were not sufficiently abundant in the recordings to quantify foraging and attack rates. Only fish that were in view inside the quadrat for at least 10 s were included. If fish left the quadrat and new individuals entered the quadrat, they were scored as separate individuals. Because it was not possible to identify individuals, we could not ascertain which individuals were counted repeatedly. Hence, to avoid pseudoreplication, we averaged the foraging and attack rates across individuals per quadrat and used quadrat (i.e., video recording) as the level of replication for statistical analyses.

### Fish collections

After completion of the visual fish surveys and camera recording inside each quadrat (approximately 1 to 5 h later), 6 to 7 individuals of the common triplefin were randomly selected in 2017 from each of the 32 survey quadrats and caught using handheld nets. A total of 211 common triplefins were caught from the vent (127) and control (84) sites (S4 Table) for physiological measurements. Because the common triplefin have a small territory of just a few square metres, the fish surveys, behavioural recordings, and physiological measurements of the collected fish would in many cases have covered the same individuals.

Because only the common triplefin was collected in 2017, we performed additional collections in 2018 and 2019 for the common triplefin and 3 additional species (blue-eyed and Yaldwin’s triplefins, and the crested blenny *Parablennius laticlavius*) for physiological measurements: approximately 10 to 16 individuals per species per year at controls and vents, respectively (S4 Table).

For all years, the fishes were humanely sacrificed, total length measured, and either preserved in 70% ethanol (2017), RNAlater (2018), or liquid nitrogen (2019) for physiological analysis (see below).

### Physiological measurements of fishes

For each individual of each of the 4 benthic fish species collected in each of the 3 years (see S4 Table), the stomach contents (a proxy for food intake), the liver (a proxy for energy storage) [51], and 2 gonads (a measure of reproductive investment) were removed and weighted. Stomach contents were oven-dried for 24 h at 70°C, while the liver and gonads were weighed upon removal (i.e., wet weight after blotting in tissue paper). Stomach content, liver, and gonad weight were all standardised for fish body size by dividing their weight by the respective fish total length and multiplying this by 100%. The gender of each individual was determined as well, and the sex ratio per quadrat was calculated as the number of males relative to the sum of males and females collected (i.e., excluding juveniles) multiplied by 100%.

Proteins can be used as a source of energy in addition to, or instead of lipids, and are also important to fuel somatic growth [52]. The tissue protein content was calculated for fishes collected in 2018 and 2019. For each fish, a sample of muscle tissue was oven-dried and ground to a fine powder using a ball mill. Samples were weighed into tin capsules and analysed for percentage nitrogen (%N) using a Horizon continuous flow isotope-ratio mass spectrometry (CF-IRMS) (Nu Instruments, Wrexham). Protein tissue content was calculated as %N × 6.25 [53].

The RNA:DNA ratio of muscle tissues is a commonly used indicator of energy allocation towards short-term somatic growth, responding to changes in food intake and body condition within 1 to 2 days [54, 55] and showing a strong correlation between the magnitude of ratio change and duration of starvation [56]. For each individual of each species approximately 4 mg of white muscle tissue was used for the analyses. The D7001 ZR-Duet DNA/RNA MiniPrep Kit (Zymo Research, Irvine) was used for DNA and RNA extraction. RNA samples were also treated with the E1010 DNase I Set (250 U) (Zymo Research, Irvine) with DNA Digestion Buffer (Zymo Research, Irvine) to prevent contamination from DNA into RNA samples. A Quantus Fluorometer (Promega, Madison) was used for quantification of the DNA and RNA samples. DNA and RNA values were standardised by the weight of the tissue sample.

While RNA:DNA ratios act as a proxy for energy allocation towards somatic growth, increment widths of otoliths (earbones) act as a proxy for actual somatic growth rates [57]. Otoliths are bone-like calcium carbonate accretions containing concentric growth increments that are laid down as a fish grows. In the common triplefin, growth rings are laid down on a daily basis [57]. We only had otoliths available for the common triplefin in year 2018. Lapilli otoliths were extracted from each fish, and the connective tissue was removed. Otoliths were then mounted onto glass slides using super glue. To expose the growth increments, otoliths were polished using incremental grades of lapping film (30 μm, 9 μm, and 3 μm). Polishing continued until all increments were visible, with great care taken to avoid over-polishing past the core and planar axis. For a more detailed explanation of otolith preparation, see [58]. We measured the distances between the outer 14 to 20 increments along a standardised axis (afterwards averaged per fish), allowing us to determine the relative growth rate for the final 2 to 3 weeks of each fish’s life. For 1 vent male and 1 control female, only 11 outer increments could be discerned. Because the ratio between males and females was unbalanced (control males, *N* = 5; control females, *N* = 5; vent males, *N* = 6; vent females, *N* = 2), we could not analyse increment width separately for the different sexes and could not include them in the SEM (i.e., too few replicates).

The cellular stress response of the fishes was evaluated by assessing the total antioxidant capacity (TAC) and malondialdehyde (MDA) production (a specific end product of lipid peroxidation), which reflect antioxidant defence and oxidative stress, respectively [59]. Oxidative stress can jeopardise cellular integrity and interfere with cellular processes and physiological traits (e.g., growth, reproduction, motility, and digestion), but can be minimised by the production of various antioxidants [60]. For each individual of each species, a piece of white muscle tissue was used to prepare a 10% tissue homogenate in an ice bath. The Coomassie Blue staining method was used to measure total protein concentration in the tissue homogenate and the absorbance (optical density, OD) was measured at 595 nm with a Jenway 6405 scanning spectrophotometer (Cole-Parmer, Staffordshire). Protein concentration (conc.) was calculated as follows:

$$Protein\;conc.(g/L)=\frac{{OD}_{sample}-{OD}_{blank}}{{OD}_{standard}-{OD}_{blank}}\times Standard\;conc.(0.563\;g/L)$$

Assay kits from Nanjing Jiancheng Bioengineering Institute (China) were used to evaluate TAC (CAT no: A015-1) and MDA concentrations (CAT no: A003-1), following the manufacturer’s manuals. OD was measured at 520 nm and 532 nm for TAC and MDA, respectively, and the levels of these biomarkers were calculated as follows:

$$\begin{array}{c} Tissue\;TAC\;content\\ \left(U/mg\;protein\right) \end{array}=\frac{{OD}_{sample}-{OD}_{contrast}}{0.01}÷30\times \begin{array}{c} volume\;of\\ homogenate \end{array}÷\begin{array}{c} Protein\;conc.\\ (mg\;protein/ml) \end{array}$$

$$\begin{array}{c} Tissue\;MDA\;content\\ \left(nmol/mg\;protein\right) \end{array}=\frac{{OD}_{sample}-{OD}_{contrast}}{{OD}_{standard}-{OD}_{blank}}\times \begin{array}{c} Standard\;conc.\\ (10\;nmol/ml) \end{array}÷\begin{array}{c} Protein\;conc.\\ (mg\;protein/ml) \end{array}$$

### Food abundance in situ

Benthic core samples (26 at controls and 25 at vents) were collected in 2017 by randomly placing a plastic jar with a 4.25-cm diameter on top of the substratum and using a spatula to slice the algae off the substratum in such way that the spatula always covered the open end of the jar to prevent any loss of invertebrate prey. The total wet biomass of turf algae was determined for each core. For a subset of the cores (10 at controls and 9 at vents), the number of gastropods and amphipods were counted (which were the main or sole prey taxa found in the cores), as well as total wet weight of the gastropods per core.

### Statistical analyses

Differences in gonad weight between controls and CO_2_ vents of the common triplefin were tested on a large set of samples (*N* = 249 individuals across 3 years, excluding 2 fishes whose tissue samples were lost; S4 Table). This univariate comparison was performed with ANOVA. To acquire insight into whether such differences also occurred across a suite of codependent physiological measures, for this species and the other 3 species, a comparative analysis was done on a much smaller dataset given the smaller availability of samples (*N* = 20 to 26 individuals per species across 2 years; S4 Table). These latter measures were derived from the same individuals. Hence, MANOVA tested for differences in energy storage (liver weight), protein content, growth (RNA:DNA ratio), antioxidant defence, oxidative damage, and food intake (stomach content weight). Reproduction was the prime focus of this study (large dataset of gonad weight, analysed by ANOVA) with supporting analysis of potential codependent measures (small dataset analysed by MANOVA). Note that the inclusion of gonad weight from the small dataset in the MANOVA would not reveal insights into how these potential codependent measures vary independently of gonad weight, i.e., the hypothesis that vents differ from controls. Because the physiological variables were measured on different scales, they were first standardised to a common scale before performing the MANOVAs. For each data point of a variable, the mean (across all data points of that variable) was subtracted and then divided by the standard deviation of the respective variable; this was done separately for each variable. For the above ANOVAs and MANOVAs, fixed factors were treatment (control versus CO_2_ vent) and gender (juvenile, male, and female), and the random factor was site (north versus south, nested within treatment).

Differences in the relative abundance of breeding males, the size–frequency distribution of the common triplefin, sex ratios, standardised gonad and liver weights, tissue protein content, tissue RNA:DNA ratios, tissue TAC and MDA concentrations, primary production, feeding rates, attacks rates, and stomach content weights were also compared between controls and vents using ANOVA. Differences in prey abundances (benthic amphipods and gastropods) were compared between controls and vents using MANOVA because they were sampled from the same cores. S1 and S2 Tables provide details of which combination of factors were considered for each analysis. All analyses were performed with the programme Primer-e (Quest Research Limited, Auckland), version 7 [61]. See S1 Data for the underlying data of the figures.

### Structural equation models (SEMs)

Our data were acquired through 2 different and independent approaches: measured within natural habitats (in situ) and measured within the laboratory (organismal physiological traits). Hence, we had to construct 2 separate SEMs, which were linked afterwards. First, to evaluate the causal relationships in our hypothesised interaction model of elevated CO_2_ effects on primary producer biomass and its cascading effects on food availability and averaged food intake by each individual fish species, we fitted individual linear models which were then aggregated in a piecewise SEM (see S5 Table). Second, we assessed the effects of CO_2_ enrichment and individual-based food intake on reproduction (gonad weight), energy storage (liver weight), growth (RNA:DNA ratios), body condition (protein content), and physiological maintenance (anti-oxidative defence and oxidative damage) for females and males of each fish species, by fitting individual linear models which were then aggregated in a piecewise SEM (see S7 Table).

Piecewise SEMs are a powerful tool that is able to estimate indirect and direct effects as well as causal links within complex networks [62]. Different from traditional SEMs, piecewise SEMs are capable of including nested models, random effects, and non-normal distributions and are less dependent on large sample sizes [62,63]. Our baseline model for food intake by fishes (see S5 Table) was constructed taking the known relationships between all measured variables into account. Thus, we specifically predicted a direct effect of CO_2_ enrichment on primary production (turf biomass), a direct effect of primary production on food abundance (gastropod prey), and a direct effect of food abundance on food intake by females and males of each individual fish species, which when taken together revealed the indirect effect of CO_2_ enrichment on food intake by fishes. We also incorporated the direct effect of CO_2_ on food abundance and food intake. The baseline model did not indicate any other missing pathways (S5 Table) and hence could be used as the final model.

The baseline model of individual physiological responses of fishes was hypothesised as being a direct effect of CO_2_ enrichment and food intake. In cases where the *p*-value of the goodness of fit test for the baseline piecewise SEM was *p* < 0.05 (S6 Table), missing pathways were identified by the model. These pathways were included in the final model (S7 Table). All relationships evaluated in the pathway analysis were based on linear models with all data square root transformed prior to model fitting.

Linear models were fitted using the package *stats* [64], and their assumptions were tested using the functions *qqPlot* (distribution of studentised residuals), *crPlots* (to evaluate the nonlinearity component), and *ncvTest* (to evaluate homoscedasticity using a nonconstant error variance test) using the package *car* [65] and validated by using the *gvlma* function (global validation test of linear model assumptions) from the *gvlma* package [66]. The piecewise SEM were generated using the package *piecewiseSEM* [67].

## Supporting information

S1 Fig Growth, demography, and sex ratio in fishes from controls and vents. (**a**) Mean otolith increment width (proxy for somatic growth in last few weeks before capture) for male (filled markers) and female (clear markers) common triplefin at control (solid line) and vent (dotted line) sites with fitted regression lines and R^2^ values. (**b**) Mean (+ SE) in situ size–abundance distribution of combined females/juveniles of the common triplefin at control and vent sites. (**c**) Mean (+ SE) sex ratios of the 4 species at controls and vents. Circles represent replicate transects (jittered on *x*-axis where values overlap; jittering on *y*-axis for sex ratio common triplefin at vents: top circles, all 100%). Sex ratio was calculated as the number of males relative to total number of males + females; gender was determined in the laboratory and included the crested blenny. See S1 Table for statistical results and S1 Data for the underlying data. ns, not significant.
(PDF)

S2 Fig Physiological proxies for fishes from controls and vents. Mean (+ SE) reproductive investment (a, b; measured as total gonad weight standardised by fish total length), energy storage (c, d; measured as liver weight standardised by fish total length), muscle tissue protein content (e, f), short-term growth (g, h; measured as muscle tissue RNA:DNA ratios), cellular antioxidant defence (i, j; measured as muscle tissue total antioxidant capacity), and cellular oxidative damage (k, l; measured as muscle tissue malondialdehyde levels) of fishes collected from controls and CO_2_ vents, for females (a, c, e, g, i, k) and males (b, d, f, h, j, l) for 3 benthic fish species. None of the physiological measurements differed between controls and vents for any of the 3 species. See S1 Table for statistical results and S1 Data for the underlying data.
(PDF)

S3 Fig Prey biomass (benthic gastropods) as a function of primary production (benthic turf biomass) at controls and CO_2_ vents. Fitted power regression line and associated *R*^*2*^- and *p*-values are included. See S1 Data for the underlying data.
(PDF)

S4 Fig In situ attack rates between mature males of the common triplefin. Attack rates are shown as a function of (a) total fish density (all benthic fish species included) at the start of the video recordings, and (b) total number of mature males of the common triplefin observed throughout the 10-min recordings. Fitted linear regression lines and associated *R*^*2*^- and *p*-values are included. See S1 Data for the underlying data.
(PDF)

S5 Fig **Mean (+ SE) in situ feeding rates (a), in situ intraspecific aggression (b), and total stomach content weights (c–e) of male and female/juvenile crested blenny, blue-eyed, and Yaldwin’s triplefins, respectively, at controls and CO**_**2**_ **vents.** The total stomach content weight is standardised as a function of fish size. Foraging and aggression data are from year 2017, while food intake data are from 2018 and 2019; no in situ foraging and aggression data could be obtained for the crested blenny in 2017. No significant CO_2_ effects were observed for the graphs above, except for a lower stomach content weight at vents for the blue-eyed triplefin (* *p* < 0.05; see S2 Table). See S1 Data for the underlying data.
(PDF)

S6 Fig Piecewise SEM for fishes at controls vs vents. The model explores (1) the direct and indirect effects of CO_2_ enrichment on primary production (turf biomass), food abundance, and food intake by fishes (top 2 levels within each species) and (2) the effects of CO_2_ enrichment and food intake on reproduction, energy storage, growth, body condition, and physiological maintenance (anti-oxidative defence TAC, and oxidative damage MDA) of fishes (bottom level for each species) for male and female common triplefins (**A**, **B**), crested blenny (**C**, **D**), blue-eyed triplefin (**E**, **F**), and Yaldwyn’s triplefin (**G**, **H**). Arrows represent standardised unidirectional relationships and arrow widths shows the strength of each interaction (see S5 and S7 Tables for estimated effects), with black arrows showing positive relationships and red arrows negative ones. The *n* (sum of fishes from controls and vents) and *R*^2^ (degree of variance explained by the linear regressions) for significant pathways for each individual linear model are also shown; arrows and R^2^ for nonsignificant pathways (*p* > 0.05) were omitted. MDA, malondialdehyde; SEM, structural equation model; TAC, total antioxidant capacity.
(PDF)

S7 Fig **Sex ratio and standardised gonad weight (i.e., divided by total fish length) as a function of water pH (a, b) and water temperature (c, d), respectively, for common triplefins (year 2017).** Fitted linear regression lines with their *R*^2^- and *p*-values are shown. Water pH and temperature were measured from within the same quadrats as fishes for physiological measurements. See S1 Data for the underlying data.
(PDF)

S1 Table MANOVAs (labelled as such) and ANOVAs (all other analyses) supporting data analysis for Figs 2 and 3 and S1 and S2 Figs. “tr” = CO_2_ treatment (control, CO_2_ vent), “si” = site (north vs south), “ge” = gender (female, male, juvenile), “ye” = year (2017, 2018, 2019; or 2018, 2019), “siz” = fish size class (in cm). Significant main effects or significant higher-order interactions (followed by post hoc pairwise tests) are indicated in bold. ANOVA, analysis of variance; MANOVA, multivariate analysis of variance.
(XLSX)

S2 Table MANOVAs (labelled as such) and ANOVAs (all other analyses) supporting data analysis for Fig 4 and S5 Fig. “tr” = CO_2_ treatment (control, CO_2_ vent), “si” = site (north vs south), “ge” = gender (female, male, juvenile). Benthic prey abundances (amphipods and gastropods) were tested with MANOVA. Significant main effects and significant higher-order interactions (followed by post-hoc pairwise tests) are indicated in bold. ANOVA, analysis of variance; MANOVA, multivariate analysis of variance.
(XLSX)

S3 Table Physicochemical measurements and concentrations of dissolved trace elements in seawater from control and CO_2_ vent fish quadrats at White Island during 2017–2019. TA, total alkalinity.
(XLSX)

S4 Table Sample sizes at controls and vents across years for each fish species. Numbers represent in situ quantification of maturity (total number of mature males evaluated) and physiological measurements in the laboratory (total number fish collected for gonads, livers, protein content, RNA:DNA ratio, antioxidant capacity, oxidative damage, and stomach content) and for in situ behavioural observations (number of videos).
(XLSX)

S5 Table Piecewise SEM coefficients. Coefficients are shown for each pathway, and correlated error structures based on the independent effects of CO_2_ enrichment on primary producer (“turf”) biomass, food abundance (gastropod prey), and food intake for male and female common triplefin, crested blenny, blue-eyed triplefin, and Yaldwyn’s triplefin. The sample size (*N*), adjusted model *R*^2^, and other specifications of each LM used in the piecewise SEM are also provided. Fisher C is a test of the conditional independence of the model and any missing pathways that should be added to the model, with the model significance (Model p) indicating missing pathways (missing if p *<* 0.05). AIC of the individual and combined effects of CO_2_ enrichment and food abundance on food intake. Significant *p*-values are indicated in bold. AIC, Akaike information criterion; LM, linear model; SEM, structural equation model.
(PDF)

S6 Table Piecewise SEM specifications for the baseline model of females and males of the common triplefin, crested blenny, blue-eyed triplefin, and Yaldwyn’s triplefin. Fisher C tests the conditional independence of the model and missing paths that should be added to the model (if model *p* < 0.05); AIC of the individual and combined effects of CO_2_ enrichment and food intake on reproduction (gonad weight), energy storage (liver weight), growth (RNA:DNA ratios), physiological maintenance (anti-oxidative defence TAC and oxidative damage MDA), and body condition (protein content). Significant *p*-values are indicated in bold. AIC, Akaike information criterion; MDA, malondialdehyde; SEM, structural equation model; TAC, total antioxidant capacity.
(PDF)

S7 Table Piecewise SEM coefficients for each pathway, correlated error, and structure specifications of each final model for male and female common triplefins, crested blennies, blue-eyed triplefins, and Yaldwyn’s triplefins. Fisher C is a test of the conditional independence of the model and any missing pathways that should be added to the model, with the model significance (Model p) indicating missing pathways (missing if *p* < 0.05). AIC of the individual and combined effects of CO_2_ enrichment and food intake, and other missing pathways as identified by the model, on reproduction (gonad weight), energy storage (liver weight), growth (RNA:DNA ratios), physiological maintenance (anti-oxidative defence TAC and oxidative damage MDA), and body condition (protein content). Significant *p*-values are indicated in bold. AIC, Akaike information criterion; MDA, malondialdehyde; SEM, structural equation model; TAC, total antioxidant capacity.
(PDF)

S1 Data Original data used to generate the figures. (XLSX)

## Abbreviations

ANOVA: analysis of variance
HCl: hydrochloric acid
ICP-MS: inductively coupled plasma mass spectrometer
IRMS: isotope-ratio mass spectrometry
MANOVA: multivariate analysis of variance
MDA: malondialdehyde
OD: optical density
*p*CO_2_: partial pressure of carbon dioxide
PVC: polyvinyl chloride
RCP: representative concentration pathway
SEM: structural equation model
TAC: total antioxidant capacity
