# Supplementary figures and images for: Ocean acidification boosts reproduction in fish via indirect effects
Source: PLoS Biol. 2021 Jan 19;19(1):e3001033. doi: 10.1371/journal.pbio.3001033 (PMC7815143; doi:10.1371/journal.pbio.3001033)

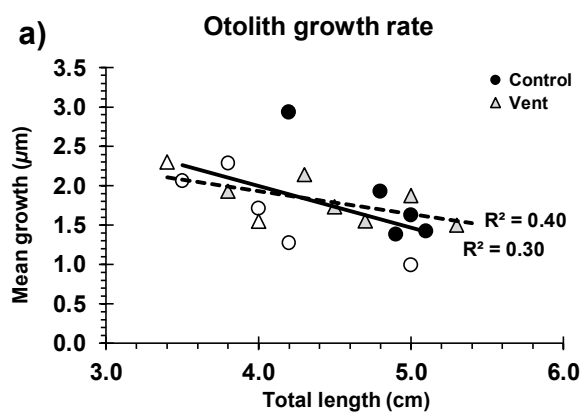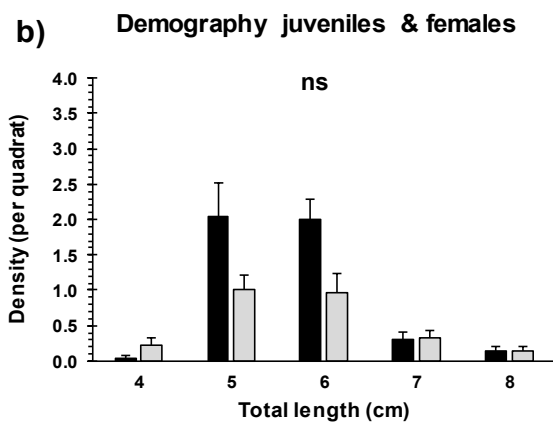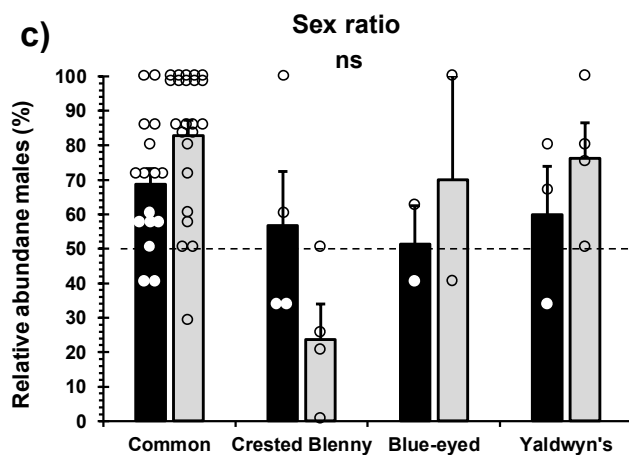

Supplement: S1 Fig — (a) Mean otolith increment width (proxy for somatic growth in last few weeks before capture) for male (filled markers) and female (clear markers) common triplefin at control (solid line) and vent (dotted line) sites with fitted regression lines and R2 values. (b) Mean (+ SE) in situ size–abundance distribution of combined females/juveniles of the common triplefin at control and vent sites. (c) Mean (+ SE) sex ratios of the 4 species at controls and vents. Circles represent replicate transects (jittered on x-axis where values overlap; jittering on y-axis for sex ratio common triplefin at vents: top circles, all 100%). Sex ratio was calculated as the number of males relative to total number of males + females; gender was determined in the laboratory and included the crested blenny. See S1 Table for statistical results and S1 Data for the underlying data. ns, not significant. (PDF) [file pbio.3001033.s001.pdf]

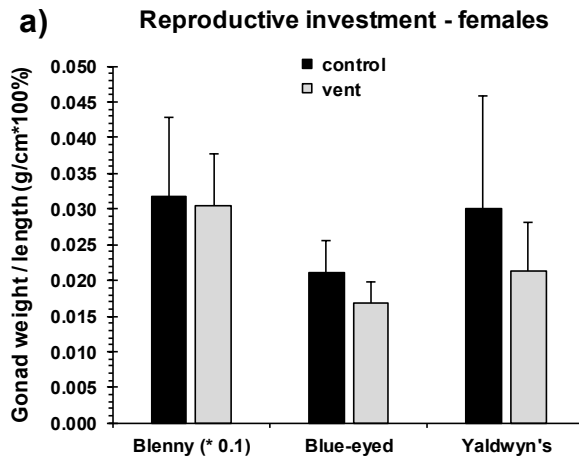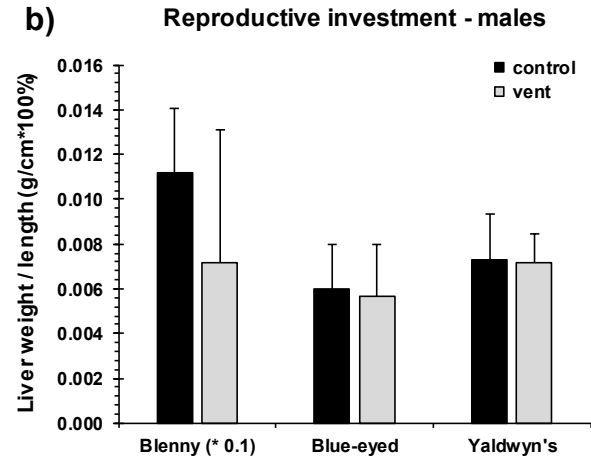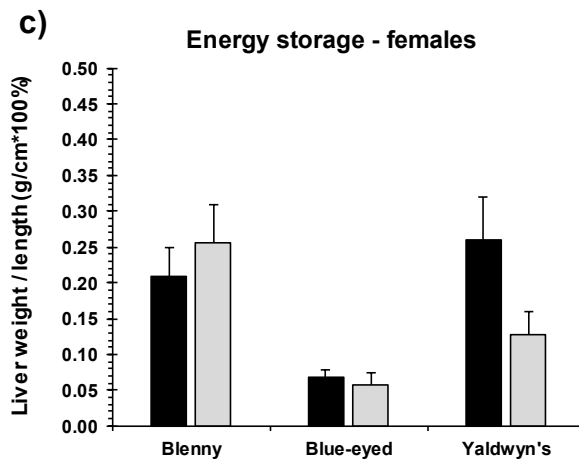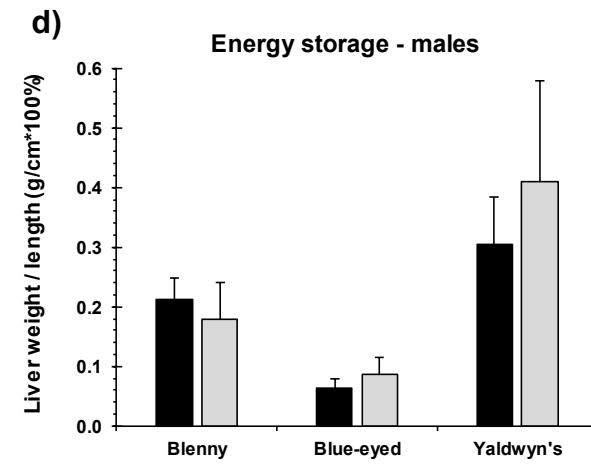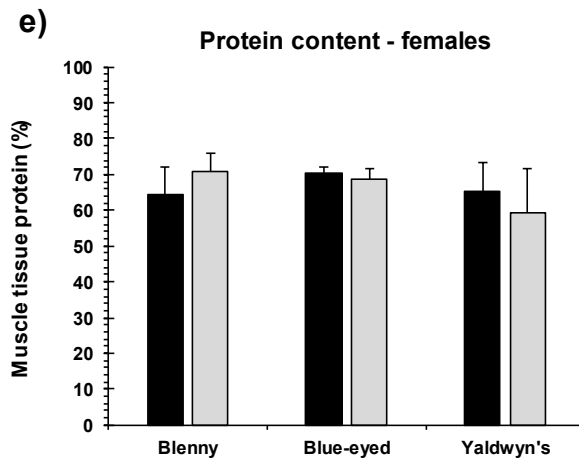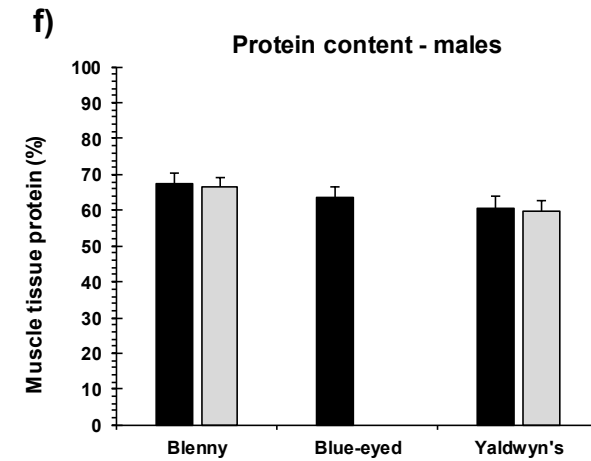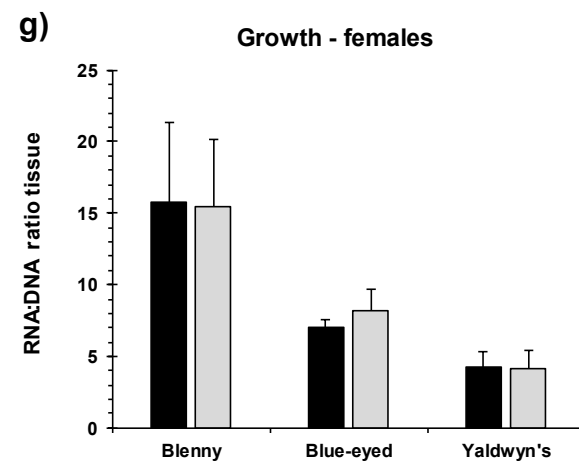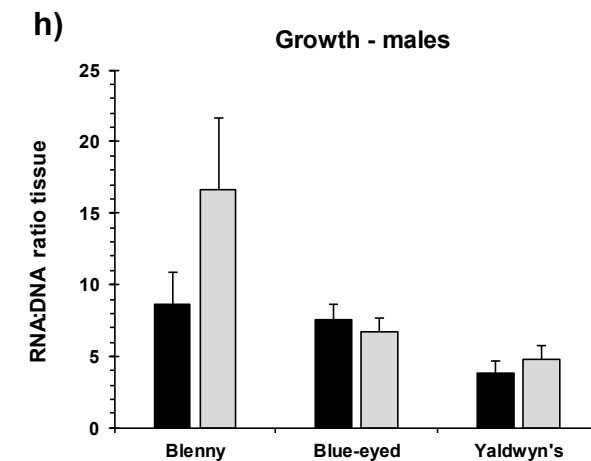

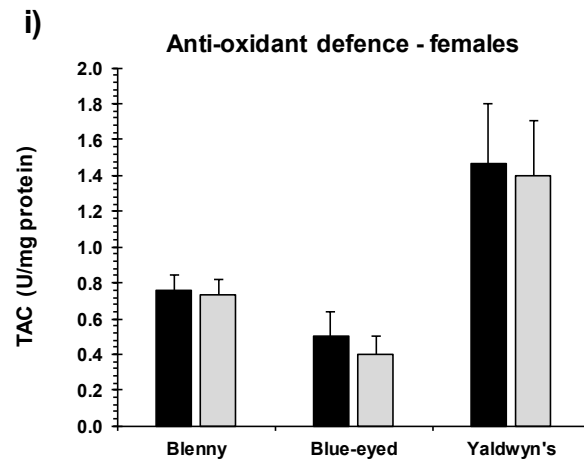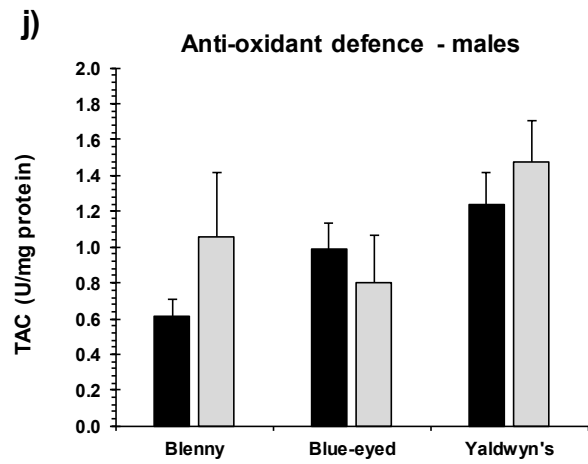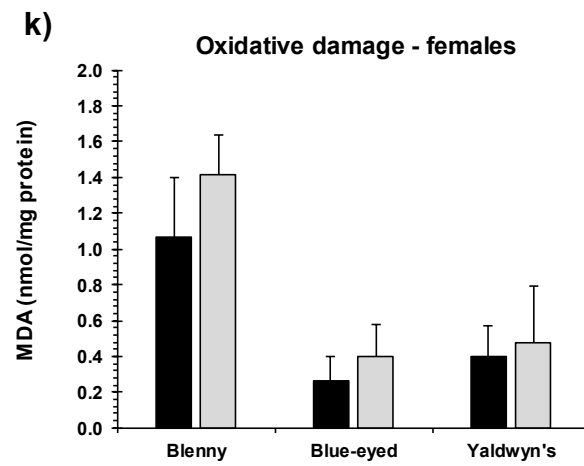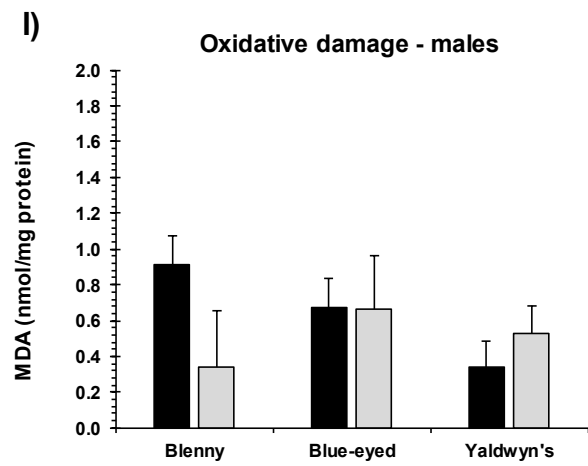

Supplement: S2 Fig — Mean (+ SE) reproductive investment (a, b; measured as total gonad weight standardised by fish total length), energy storage (c, d; measured as liver weight standardised by fish total length), muscle tissue protein content (e, f), short-term growth (g, h; measured as muscle tissue RNA:DNA ratios), cellular antioxidant defence (i, j; measured as muscle tissue total antioxidant capacity), and cellular oxidative damage (k, l; measured as muscle tissue malondialdehyde levels) of fishes collected from controls and CO2 vents, for females (a, c, e, g, i, k) and males (b, d, f, h, j, l) for 3 benthic fish species. None of the physiological measurements differed between controls and vents for any of the 3 species. See S1 Table for statistical results and S1 Data for the underlying data. (PDF) [file pbio.3001033.s002.pdf]

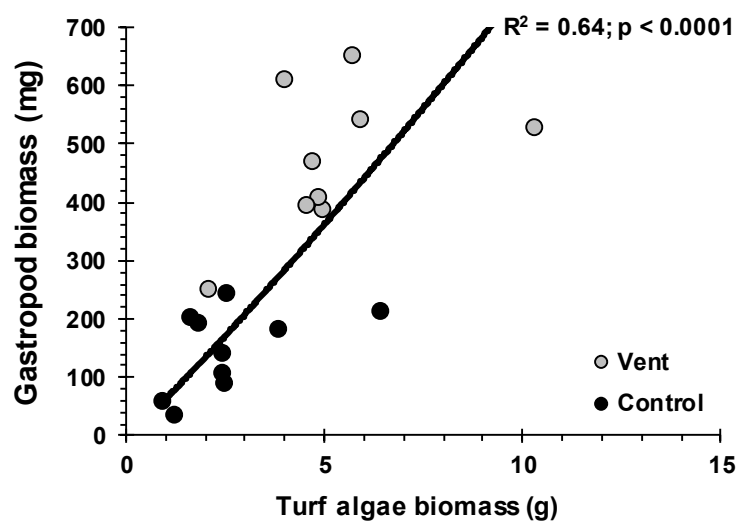

Supplement: S3 Fig — Fitted power regression line and associated R2- and p-values are included. See S1 Data for the underlying data. (PDF) [file pbio.3001033.s003.pdf]

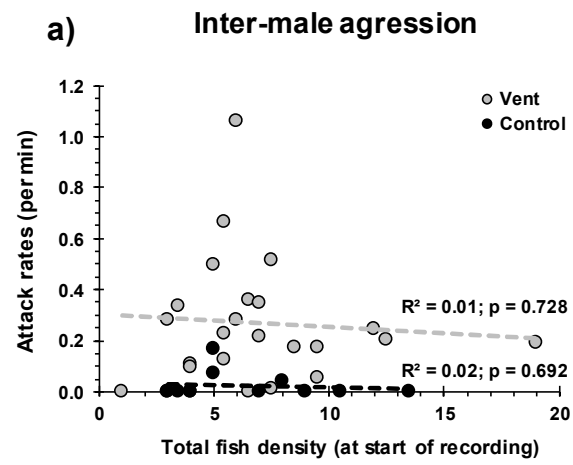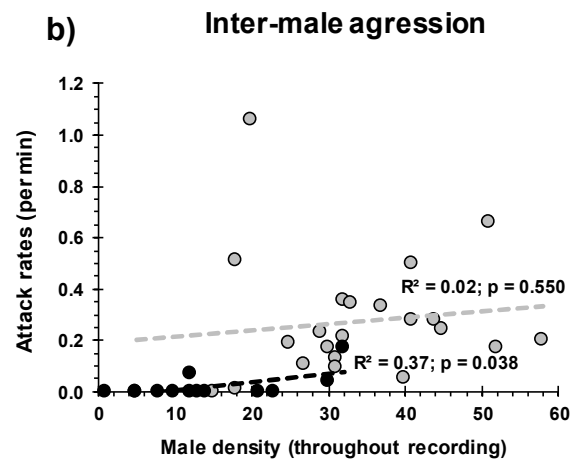

Supplement: S4 Fig — Attack rates are shown as a function of (a) total fish density (all benthic fish species included) at the start of the video recordings, and (b) total number of mature males of the common triplefin observed throughout the 10-min recordings. Fitted linear regression lines and associated R2- and p-values are included. See S1 Data for the underlying data. (PDF) [file pbio.3001033.s004.pdf]

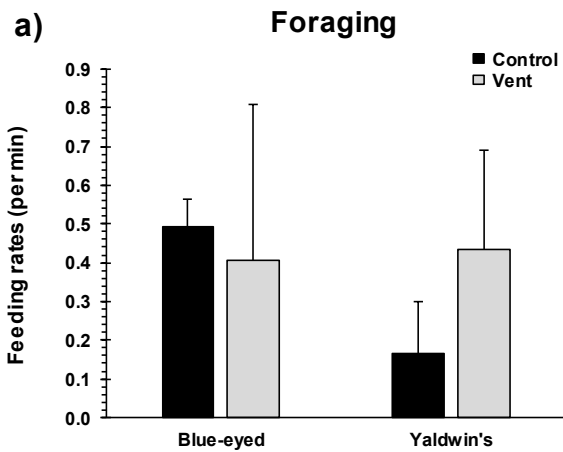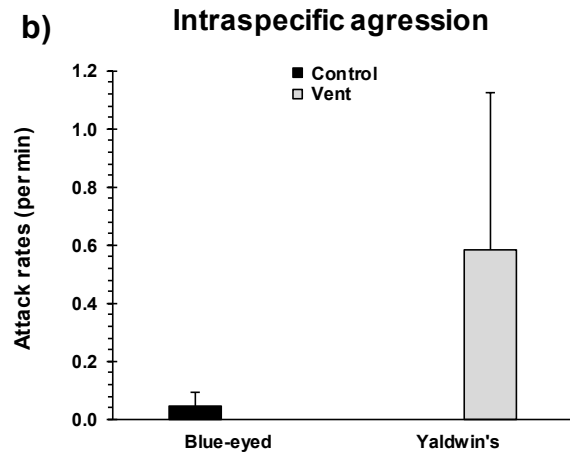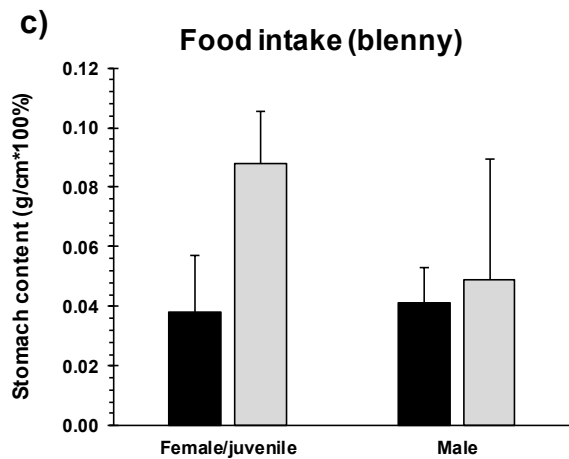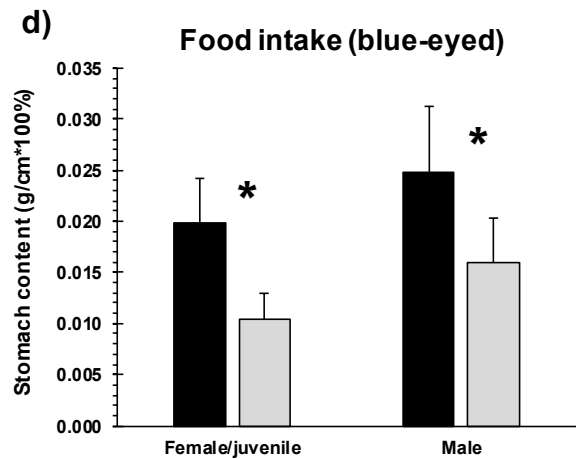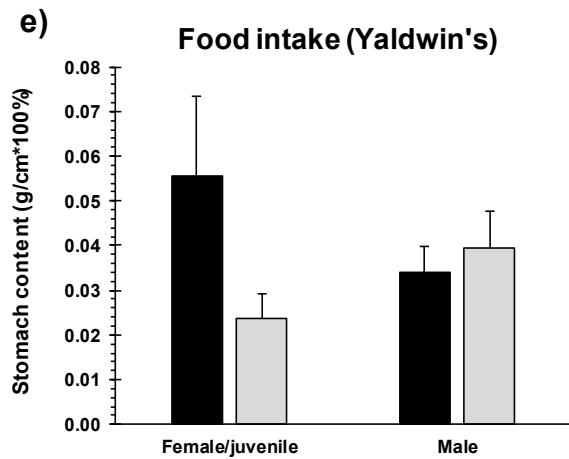

Supplement: S5 Fig — Mean (+ SE) in situ feeding rates (a), in situ intraspecific aggression (b), and total stomach content weights (c–e) of male and female/juvenile crested blenny, blue-eyed, and Yaldwin’s triplefins, respectively, at controls and CO2 vents. The total stomach content weight is standardised as a function of fish size. Foraging and aggression data are from year 2017, while food intake data are from 2018 and 2019; no in situ foraging and aggression data could be obtained for the crested blenny in 2017. No significant CO2 effects were observed for the graphs above, except for a lower stomach content weight at vents for the blue-eyed triplefin (* p < 0.05; see S2 Table). See S1 Data for the underlying data. (PDF) [file pbio.3001033.s005.pdf]

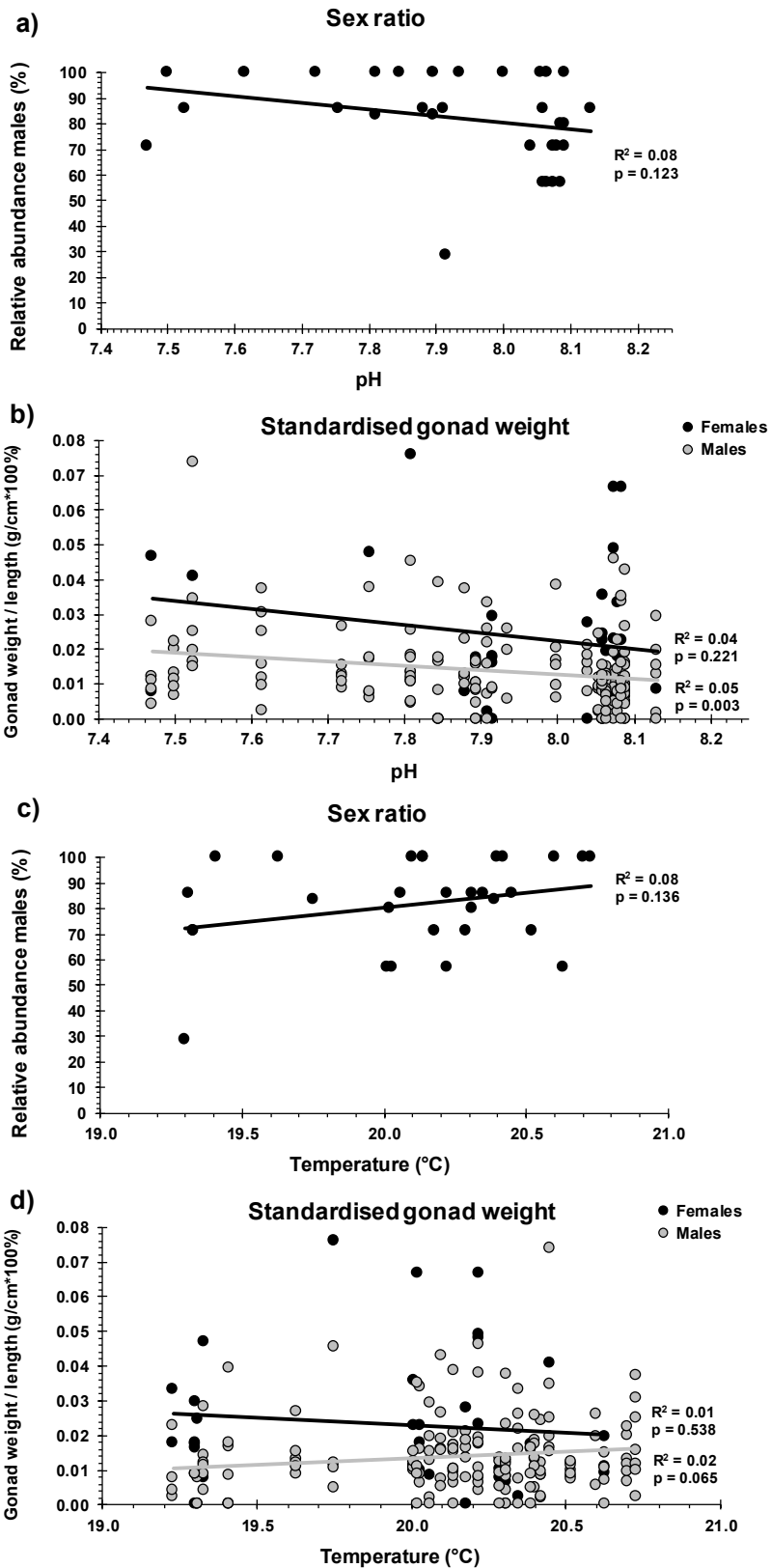

Supplement: S7 Fig — Sex ratio and standardised gonad weight (i.e., divided by total fish length) as a function of water pH (a, b) and water temperature (c, d), respectively, for common triplefins (year 2017). Fitted linear regression lines with their R2- and p-values are shown. Water pH and temperature were measured from within the same quadrats as fishes for physiological measurements. See S1 Data for the underlying data. (PDF) [file pbio.3001033.s007.pdf]
