# Supplementary material for: Ocean acidification boosts reproduction in fish via indirect effects
Source: PLoS Biol. 2021 Jan 19;19(1):e3001033. doi: 10.1371/journal.pbio.3001033 (PMC7815143; doi:10.1371/journal.pbio.3001033)
Supplement: S6 Fig — The model explores (1) the direct and indirect effects of CO2 enrichment on primary production (turf biomass), food abundance, and food intake by fishes (top 2 levels within each species) and (2) the effects of CO2 enrichment and food intake on reproduction, energy storage, growth, body condition, and physiological maintenance (anti-oxidative defence TAC, and oxidative damage MDA) of fishes (bottom level for each species) for male and female common triplefins (A, B), crested blenny (C, D), blue-eyed triplefin (E, F), and Yaldwyn’s triplefin (G, H). Arrows represent standardised unidirectional relationships and arrow widths shows the strength of each interaction (see S5 and S7 Tables for estimated effects), with black arrows showing positive relationships and red arrows negative ones. The n (sum of fishes from controls and vents) and R2 (degree of variance explained by the linear regressions) for significant pathways for each individual linear model are also shown; arrows and R2 for nonsignificant pathways (p > 0.05) were omitted. MDA, malondialdehyde; SEM, structural equation model; TAC, total antioxidant capacity. (PDF) [file pbio.3001033.s006.pdf]

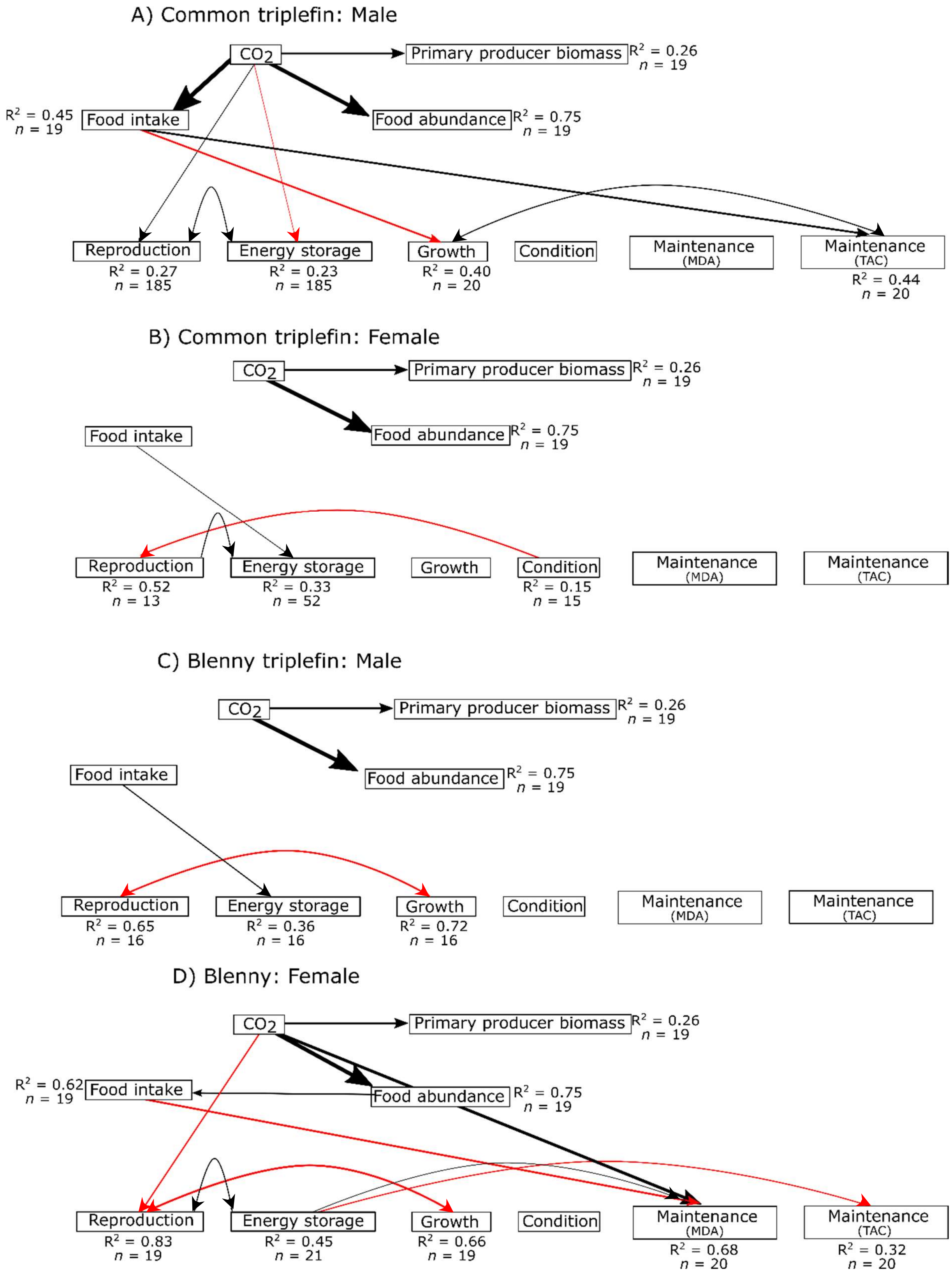
A) Common triplefin: Male
CO2
Primary producer biomass
R2 = 0.26
n = 19
R2 = 0.45
n = 19
Food intake
Food abundance
R2 = 0.75
n = 19
Reproduction
Energy storage
Growth
Condition
Maintenance (MDA)
Maintenance (TAC)
R2 = 0.27
n = 185
R2 = 0.23
n = 185
R2 = 0.40
n = 20
R2 = 0.44
n = 20
B) Common triplefin: Female
CO2
Primary producer biomass
R2 = 0.26
n = 19
Food intake
Food abundance
R2 = 0.75
n = 19
Reproduction
Energy storage
Growth
Condition
Maintenance (MDA)
Maintenance (TAC)
R2 = 0.52
n = 13
R2 = 0.33
n = 52
R2 = 0.15
n = 15
C) Blenny triplefin: Male
CO2
Primary producer biomass
R2 = 0.26
n = 19
Food intake
Food abundance
R2 = 0.75
n = 19
Reproduction
Energy storage
Growth
Condition
Maintenance (MDA)
Maintenance (TAC)
R2 = 0.65
n = 16
R2 = 0.36
n = 16
R2 = 0.72
n = 16
D) Blenny: Female
CO2
Primary producer biomass
R2 = 0.26
n = 19
R2 = 0.62
n = 19
Food intake
Food abundance
R2 = 0.75
n = 19
Reproduction
Energy storage
Growth
Condition
Maintenance (MDA)
Maintenance (TAC)
R2 = 0.83
n = 19
R2 = 0.45
n = 21
R2 = 0.66
n = 19
R2 = 0.68
n = 20
R2 = 0.32
n = 20

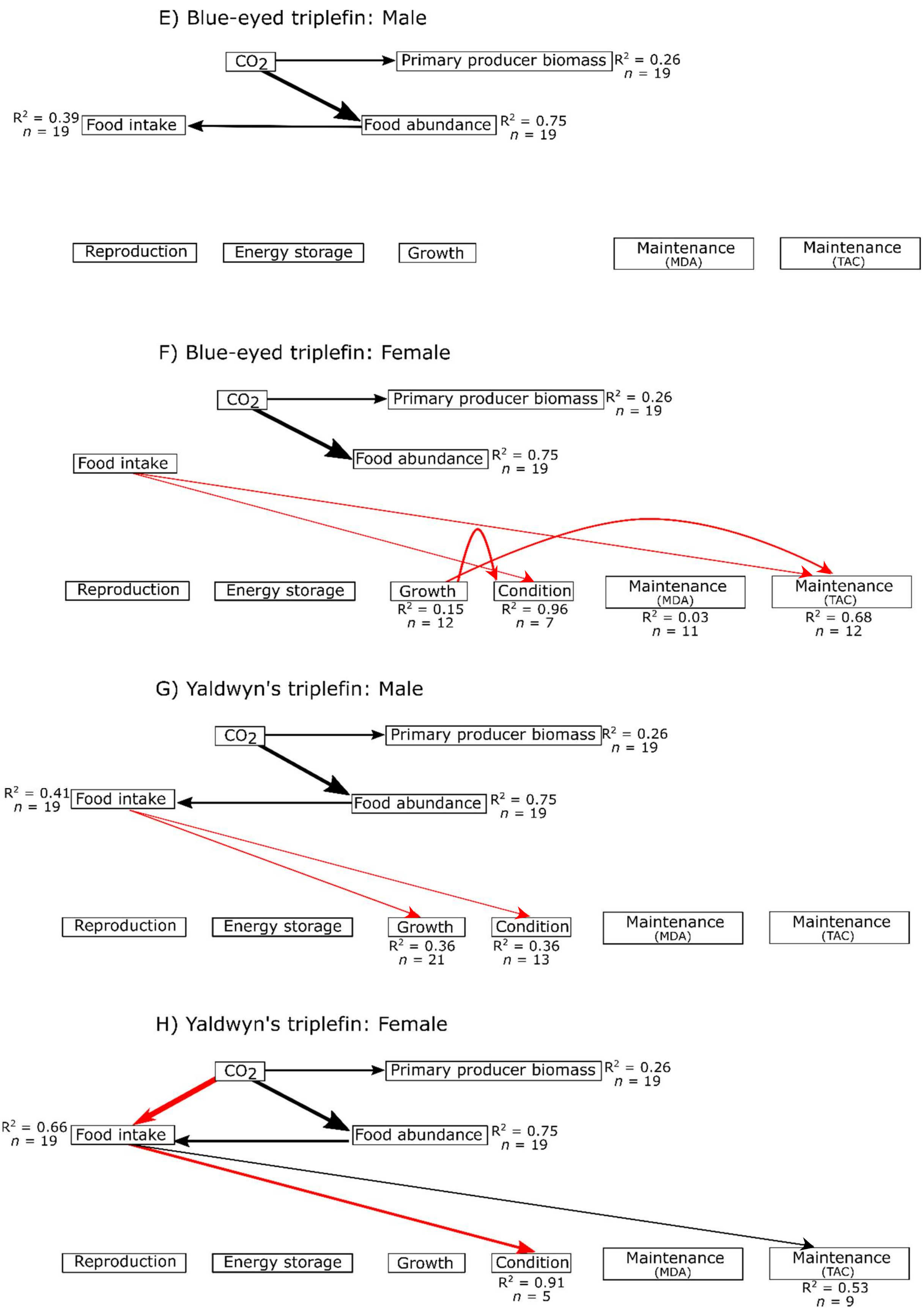
E) Blue-eyed triplefin: Male
CO2
Primary producer biomass
R² = 0.26
n = 19
R² = 0.39
n = 19
Food intake
Food abundance
R² = 0.75
n = 19
Reproduction
Energy storage
Growth
Maintenance
(MDA)
Maintenance
(TAC)
F) Blue-eyed triplefin: Female
CO2
Primary producer biomass
R² = 0.26
n = 19
Food intake
Food abundance
R² = 0.75
n = 19
Reproduction
Energy storage
Growth
R² = 0.15
n = 12
Condition
R² = 0.96
n = 7
Maintenance
(MDA)
R² = 0.03
n = 11
Maintenance
(TAC)
R² = 0.68
n = 12
G) Yaldwyn's triplefin: Male
CO2
Primary producer biomass
R² = 0.26
n = 19
R² = 0.41
n = 19
Food intake
Food abundance
R² = 0.75
n = 19
Reproduction
Energy storage
Growth
R² = 0.36
n = 21
Condition
R² = 0.36
n = 13
Maintenance
(MDA)
Maintenance
(TAC)
H) Yaldwyn's triplefin: Female
CO2
Primary producer biomass
R² = 0.26
n = 19
R² = 0.66
n = 19
Food intake
Food abundance
R² = 0.75
n = 19
Reproduction
Energy storage
Growth
Condition
R² = 0.91
n = 5
Maintenance
(MDA)
Maintenance
(TAC)
R² = 0.53
n = 9
