# Supplementary material for: Ocean acidification boosts reproduction in fish via indirect effects
Source: PLoS Biol. 2021 Jan 19;19(1):e3001033. doi: 10.1371/journal.pbio.3001033 (PMC7815143; doi:10.1371/journal.pbio.3001033)
Supplement: S5 Table — Coefficients are shown for each pathway, and correlated error structures based on the independent effects of CO2 enrichment on primary producer (“turf”) biomass, food abundance (gastropod prey), and food intake for male and female common triplefin, crested blenny, blue-eyed triplefin, and Yaldwyn’s triplefin. The sample size (N), adjusted model R2, and other specifications of each LM used in the piecewise SEM are also provided. Fisher C is a test of the conditional independence of the model and any missing pathways that should be added to the model, with the model significance (Model p) indicating missing pathways (missing if p < 0.05). AIC of the individual and combined effects of CO2 enrichment and food abundance on food intake. Significant p-values are indicated in bold. AIC, Akaike information criterion; LM, linear model; SEM, structural equation model. (PDF) [file pbio.3001033.s012.pdf]

Species	Sex	Response variable	Predictor variables	Model	N	Estimate	SE	p	R^2	Fisher C	Model p	AIC
		Turf biomass	CO_2 enrichment	LM	19	0.9849	0.4080	**0.0273**	0.26			
		Food abundance	CO_2 enrichment	LM	19	1.6435	0.2835	**<0.0001**	0.75			
			Turf biomass			0.0415	0.1455	0.7789				
Common	Male	Food intake	CO_2 enrichment	LM	19	2.2852	0.7167	**0.0057**	0.45	8.78	0.186	24.82
			Food abundance			-0.6909	0.3677	0.0786				
	Female	Food intake	CO_2 enrichment	LM	19	-0.9485	0.9319	0.3239	0.07	4.66	0.588	23.33
			Food abundance			0.3043	0.4780	0.5334				
Blenny	Male	Food intake	CO_2 enrichment	LM	19	1.1591	0.6133	0.0770	0.60	4.66	0.588	23.33
			Food abundance			0.1997	0.3146	0.5345				
	Female	Food intake	CO_2 enrichment	LM	19	0.1927	0.6004	0.7524	0.62	7.81	0.252	25.09
			Food abundance			0.6975	0.3080	**0.0378**				
Blue-eyed	Male	Food intake	CO_2 enrichment	LM	19	-1.5754	0.7561	0.0536	0.39	7.72	0.259	24.67
			Food abundance			1.1722	0.3879	**0.0081**				
	Female	Food intake	CO_2 enrichment	LM	19	0.7549	0.4586	0.1192	0.15	5.99	0.427	24.37
			Food abundance			-1.1632	0.8939	0.2116				
Yaldwyn's	Male	Food intake	CO_2 enrichment	LM	19	-1.0403	0.7439	0.1811	0.41	7.34	0.290	24.64
			Food abundance			1.0419	0.3816	**0.0148**				
	Female	Food intake	CO_2 enrichment	LM	19	-3.0863	0.5623	**<0.0001**	0.66	9.04	0.171	24.09
			Food abundance			1.2023	0.2885	**<0.0001**				
