# Supplementary material for: Ocean acidification boosts reproduction in fish via indirect effects
Source: PLoS Biol. 2021 Jan 19;19(1):e3001033. doi: 10.1371/journal.pbio.3001033 (PMC7815143; doi:10.1371/journal.pbio.3001033)
Supplement: S6 Table — Fisher C tests the conditional independence of the model and missing paths that should be added to the model (if model p < 0.05); AIC of the individual and combined effects of CO2 enrichment and food intake on reproduction (gonad weight), energy storage (liver weight), growth (RNA:DNA ratios), physiological maintenance (anti-oxidative defence TAC and oxidative damage MDA), and body condition (protein content). Significant p-values are indicated in bold. AIC, Akaike information criterion; MDA, malondialdehyde; SEM, structural equation model; TAC, total antioxidant capacity. (PDF) [file pbio.3001033.s013.pdf]

Species	Sex	Response variable	Predictor variables	Fisher C	Model p	AIC	N
Common	Male	Reproduction	Food intake	96.89	**<0.0001**	144.89	185
			CO_2 enrichment				
		Energy storage	Food intake				185
			CO_2 enrichment				
		Growth	Food intake				20
			CO_2 enrichment				
		Maintenance (TAC)	Food intake				20
			CO_2 enrichment				
		Maintenance (MDA)	Food intake				20
			CO_2 enrichment				
		Condition	Food intake				15
			CO_2 enrichment				
	Female	Reproduction	Food intake	54.13	**0.004**	102.13	13
			CO_2 enrichment				
		Energy storage	Food intake				52
			CO_2 enrichment				
		Growth	Food intake				15
			CO_2 enrichment				
		Maintenance (TAC)	Food intake				15
			CO_2 enrichment				
		Maintenance (MDA)	Food intake				15
			CO_2 enrichment				
		Condition	Food intake				13
			CO_2 enrichment				

Species	Sex	Response variable	Predictor variables	Fisher C	Model p	AIC	N
Blenny	Male	Reproduction	Food intake<br>CO_2 enrichment	36.97	0.178	84.97	16
		Energy storage	Food intake<br>CO_2 enrichment				16
		Growth	Food intake<br>CO_2 enrichment				16
		Maintenance (TAC)	Food intake<br>CO_2 enrichment				16
		Maintenance (MDA)	Food intake<br>CO_2 enrichment				16
		Condition	Food intake<br>CO_2 enrichment				13
	Female	Reproduction	Food intake<br>CO_2 enrichment	61.79	**0.001**	109.79	19
		Energy storage	Food intake<br>CO_2 enrichment				21
		Growth	Food intake<br>CO_2 enrichment				19
		Maintenance (TAC)	Food intake<br>CO_2 enrichment				19
		Maintenance (MDA)	Food intake<br>CO_2 enrichment				19
		Condition	Food intake<br>CO_2 enrichment				16

Species	Sex	Response variable	Predictor variables	Fisher C	Model p	AIC	N
Blue-eyed	Male	Reproduction	Food intake<br>CO_2 enrichment	17.98	0.589	57.98	14
		Energy storage	Food intake<br>CO_2 enrichment				14
		Growth	Food intake<br>CO_2 enrichment				13
		Maintenance (TAC)	Food intake<br>CO_2 enrichment				13
		Maintenance (MDA)	CO_2 enrichment<br>Food intake				13
	Female	Reproduction	Food intake<br>CO_2 enrichment	61.09	**0.001**	109.09	13
		Energy storage	Food intake<br>CO_2 enrichment				13
		Growth	Food intake<br>CO_2 enrichment				12
		Maintenance (TAC)	Food intake<br>CO_2 enrichment				12
		Maintenance (MDA)	Food intake<br>CO_2 enrichment				11
		Condition	Food intake<br>CO_2 enrichment				7

Species	Sex	Response variable	Predictor variables	Fisher C	Model p	AIC	N
Yaldwyn's	Male	Reproduction	Food intake<br>CO_2 enrichment	36.74	0.185	87.74	22
		Energy storage	Food intake<br>CO_2 enrichment				22
		Growth	Food intake<br>CO_2 enrichment				21
		Maintenance (TAC)	Food intake<br>CO_2 enrichment				21
		Maintenance (MDA)	Food intake<br>CO_2 enrichment				21
		Condition	Food intake<br>CO_2 enrichment				13
	Female	Reproduction	Food intake<br>CO_2 enrichment	23.97	0.773	71.97	9
		Energy storage	Food intake<br>CO_2 enrichment				9
		Growth	Food intake<br>CO_2 enrichment				8
		Maintenance (TAC)	Food intake<br>CO_2 enrichment				9
		Maintenance (MDA)	Food intake<br>CO_2 enrichment				9
		Condition	Food intake<br>CO_2 enrichment				5
