# Supplementary material for: Ocean acidification boosts reproduction in fish via indirect effects
Source: PLoS Biol. 2021 Jan 19;19(1):e3001033. doi: 10.1371/journal.pbio.3001033 (PMC7815143; doi:10.1371/journal.pbio.3001033)
Supplement: S7 Table — Fisher C is a test of the conditional independence of the model and any missing pathways that should be added to the model, with the model significance (Model p) indicating missing pathways (missing if p < 0.05). AIC of the individual and combined effects of CO2 enrichment and food intake, and other missing pathways as identified by the model, on reproduction (gonad weight), energy storage (liver weight), growth (RNA:DNA ratios), physiological maintenance (anti-oxidative defence TAC and oxidative damage MDA), and body condition (protein content). Significant p-values are indicated in bold. AIC, Akaike information criterion; MDA, malondialdehyde; SEM, structural equation model; TAC, total antioxidant capacity. (PDF) [file pbio.3001033.s014.pdf]

Species	Sex	Response variable	Predictor variables	Estimate	SD	p	R^2	Fisher C	Model p	AIC	N
			Energy storage	0.4715	0.0636	**<0.0001**					
		Reproduction	Food intake	0.1221	0.0641	0.0584	0.273				185
			CO_2 enrichment	0.3712	0.1322	**0.0055**					
			Reproduction	0.4943	0.0667	**<0.0001**					
		Energy storage	Food intake	-0.0123	0.0663	0.8532	0.239				185
			CO_2 enrichment	-0.2922	0.1366	**0.0337**					
			Maintenance (TAC)	0.5807	0.2146	**0.0156**					
	Male	Growth	Food intake	-0.8876	0.3460	**0.0207**	0.408	8.00	0.785	64	20
			CO_2 enrichment	0.5775	0.3800	0.1481					
			Food intake	0.9590	0.3159	**0.0079**					
		Maintenance (TAC)	Growth	0.5406	0.1998	**0.0156**	0.449				20
			CO_2 enrichment	-0.4677	0.3744	0.2296					
		Maintenance (MDA)	Food intake	0.0007	0.3905	0.9986	0.014				20
			CO_2 enrichment	-0.2304	0.4720	0.6317					
Common		Condition	Food intake	-0.4581	0.3954	0.2692	0.155				15
			CO_2 enrichment	0.4123	0.5169	0.4405					
			Condition	-0.7161	0.3059	**0.0474**					
		Reproduction	Energy storage	0.4451	0.2511	0.1142	0.516				13
			Food intake	-0.3582	0.2953	0.2597					
			CO_2 enrichment	-0.1214	0.6100	0.8472					
			Reproduction	0.4591	0.1189	**0.0003**					
		Energy storage	Food intake	0.3442	0.1189	**0.0057**	0.328				52
			CO_2 enrichment	-0.2041	0.2396	0.3985					
	Female	Growth	Food intake	0.0368	0.2982	0.9039	0.025	32.96	0.163	86.96	15
			CO_2 enrichment	-0.3211	0.5850	0.5932					
		Maintenance (TAC)	Food intake	0.2650	0.2779	0.3592	0.153				15
			CO_2 enrichment	-0.6399	0.5453	0.2634					
		Maintenance (MDA)	Food intake	-0.0356	0.2724	0.8982	0.187				15
			CO_2 enrichment	0.8862	0.5344	0.1232					
		Condition	Food intake	0.1874	0.2976	0.5431	0.071				13
			CO_2 enrichment	-0.3992	0.6352	0.5438					

Species	Sex	Response variable	Predictor variables	Estimate	SD	p	R^2	Fisher C	Model p	AIC	N
Blenny	Male	Reproduction	Growth	-0.8481	0.2157	**0.0020**	0.649	17.51	0.620	69.51	16
			Food intake	-0.0199	0.2020	0.9233					
			CO_2 enrichment	0.2308	0.4135	0.5870					
		Energy storage	Food intake	0.5878	0.2240	**0.0210**	0.357				16
			CO_2 enrichment	-0.1323	0.5008	0.7958					
		Growth	Reproduction	-0.6637	0.1688	**0.0020**	0.725				16
			Food intake	-0.2265	0.1664	0.1985					
			CO_2 enrichment	0.4560	0.3463	0.2125					
		Maintenance (TAC)	Food intake	-0.1004	0.2519	0.6968	0.187				16
			CO_2 enrichment	0.9136	0.5633	0.1288					
		Maintenance (MDA)	Food intake	0.4474	0.2172	0.0600	0.396				16
			CO_2 enrichment	-0.8751	0.4857	0.0948					
		Condition	Food intake	0.3453	0.2699	0.2297	0.141				13
			CO_2 enrichment	0.0763	0.6187	0.9043					
	Female	Reproduction	Growth	-0.5950	0.1150	**0.0001**	0.833	16.64	0.548	76.64	19
			Energy storage	0.2895	0.1271	**0.0390**					
			Food intake	0.2276	0.1374	0.1199					
			CO_2 enrichment	-0.6751	0.2602	**0.0212**					
		Energy storage	Reproduction	0.5374	0.2276	**0.0304**	0.454				21
			Food intake	0.2194	0.2439	0.3809					
			CO_2 enrichment	0.0486	0.4365	0.9126					
		Growth	Reproduction	-0.9756	0.1953	**0.0002**	0.665				19
			Food intake	0.2246	0.1995	0.2781					
			CO_2 enrichment	-0.7021	0.3995	0.0993					
		Maintenance (TAC)	Energy storage	-0.5609	0.2527	**0.0423**	0.319				19
			Food intake	-0.0177	0.2710	0.9488					
			CO_2 enrichment	-0.3564	0.5238	0.5066					
		Maintenance (MDA)	Food intake	-0.9075	0.1795	**0.0001**	0.684				19
			CO_2 enrichment	1.4842	0.3470	**0.0007**					
			Energy storage	0.3671	0.1674	**0.0445**					
		Condition	Food intake	0.0438	0.2534	0.8655	0.100				16
			CO_2 enrichment	0.5837	0.5570	0.3138					

Species	Sex	Response variable	Predictor variables	Estimate	SD	p	R^2	Fisher C	Model p	AIC	N
Blue-eyed	Male	Reproduction	Food intake	-0.1449	0.3034	0.6423	0.023	17.98	0.589	57.98	14
			CO_2 enrichment	0.0534	0.5847	0.9289					
		Energy storage	Food intake	0.2141	0.2922	0.4792	0.094				14
			CO_2 enrichment	0.5065	0.5632	0.3877					
		Growth	Food intake	-0.1334	0.3112	0.6773	0.047				13
			CO_2 enrichment	-0.3618	0.5992	0.5595					
		Maintenance (TAC)	Food intake	0.3737	0.2841	0.2178	0.206				13
			CO_2 enrichment	-0.4170	0.5471	0.4636					
		Maintenance (MDA)	CO_2 enrichment	-0.2783	0.6027	0.6542	0.036				13
			Food intake	0.1077	0.3129	0.7380					
	Female	Reproduction	Energy storage	0.6026	0.2706	0.0530	0.359	16.22	0.438	72.22	13
			Food intake	0.1094	0.2675	0.6921					
			CO_2 enrichment	0.1916	0.5345	0.7283					
		Energy storage	Reproduction	0.5895	0.2647	0.0530	0.373				13
			Food intake	-0.0975	0.2650	0.7214					
			CO_2 enrichment	-0.3180	0.5218	0.5573					
		Growth	Food intake	-0.2825	0.2943	0.3623	0.151				12
			CO_2 enrichment	0.4660	0.5970	0.4551					
		Maintenance (TAC)	Growth	-0.7960	0.2168	**0.0063**	0.681				12
			Food intake	-0.5629	0.2010	**0.0232**					
			CO_2 enrichment	0.0738	0.4013	0.8587					
		Maintenance (MDA)	Food intake	-0.0731	0.3454	0.8376	0.034				11
			CO_2 enrichment	0.3323	0.6665	0.6315					
		Condition	Growth	-1.1048	0.1327	**0.0036**	0.962				7
			Food intake	-0.4850	0.1334	**0.0359**					
			CO_2 enrichment	0.6494	0.2616	0.0891					

Species	Sex	Response variable	Predictor variables	Estimate	SD	p	R^2	Fisher C	Model p	AIC	N
Yaldwyn's	Male	Reproduction	Food intake	0.1536	0.2193	0.4921	0.047	36.74	0.185	87.74	22
			CO_2 enrichment	0.2911	0.4303	0.5068					
		Energy storage	Food intake	-0.1018	0.2241	0.6549	0.011				22
			CO_2 enrichment	-0.0177	0.4398	0.9683					
		Growth	Food intake	-0.6130	0.1991	**0.0065**	0.360				21
			CO_2 enrichment	0.3769	0.3808	0.3354					
		Maintenance (TAC)	Food intake	0.2506	0.2193	0.2682	0.092				21
			CO_2 enrichment	0.3359	0.4430	0.4581					
		Maintenance (MDA)	Food intake	-0.2574	0.2183	0.2538	0.101				21
			CO_2 enrichment	0.3346	0.4409	0.4577					
		Condition	Food intake	-0.5004	0.2127	**0.0405**	0.359				13
			CO_2 enrichment	-0.2615	0.5213	0.6268					
	Female	Reproduction	Food intake	-0.0725	0.4146	0.8670	0.006	23.97	0.773	71.97	9
			CO_2 enrichment	-0.0785	0.8292	0.9276					
		Energy storage	Food intake	0.2784	0.3446	0.4500	0.313				9
			CO_2 enrichment	-0.8710	0.6892	0.2532					
		Growth	Food intake	-0.7493	0.4603	0.1645	0.347				8
			CO_2 enrichment	-0.0781	0.6987	0.9154					
		Maintenance (TAC)	Food intake	0.7389	0.2861	**0.0416**	0.527				9
			CO_2 enrichment	0.2472	0.5721	0.6808					
		Maintenance (MDA)	Food intake	-0.3418	0.3917	0.4164	0.113				9
			CO_2 enrichment	-0.1065	0.7833	0.8963					
		Condition	Food intake	-1.3809	0.3163	**0.0487**	0.912				5
			CO_2 enrichment	0.4394	0.4377	0.4212					
